# Programmable integrin and N-cadherin adhesive interactions modulate mechanosensing of mesenchymal stem cells by cofilin phosphorylation

**DOI:** 10.1038/s41467-022-34424-0

**Published:** 2022-11-11

**Authors:** Zheng Zhang, Baoyong Sha, Lingzhu Zhao, Huan Zhang, Jinteng Feng, Cheng Zhang, Lin Sun, Meiqing Luo, Bin Gao, Hui Guo, Zheng Wang, Feng Xu, Tian Jian Lu, Guy M. Genin, Min Lin

**Affiliations:** 1 The Key Laboratory of Biomedical Information Engineering of Ministry of Education, School of Life Science and Technology, Xi’an Jiaotong University, Xi’an, 710049 P.R. China; 2 Bioinspired Engineering and Biomechanics Center (BEBC), Xi’an Jiaotong University, Xi’an, 710049 P.R. China; 3 School of Basic Medical Science, Xi’an Medical University, Xi’an, 710021 P.R. China; 4 Department of Medical Oncology, First Affiliated Hospital of Xi’an Jiaotong University, Xi’an, 710061 P.R. China; 5 Department of Endocrinology, Second Affiliated Hospital of Air Force Military Medical University, Xi’an, 710038 P.R. China; 6 Department of Hepatobiliary Surgery, First Affiliated Hospital of Xi’an Jiaotong University, Xi’an, 710061 P.R. China; 7 State Key Laboratory of Mechanics and Control of Mechanical Structures, Nanjing University of Aeronautics and Astronautics, Nanjing, 210016 P.R. China; 8 MIIT Key Laboratory for Multifunctional Lightweight Materials and Structures, Nanjing University of Aeronautics and Astronautics, Nanjing, 210016 P.R. China; 9 Department of Mechanical Engineering & Materials Science, Washington University in St. Louis, St. Louis, 63130 MO USA; 10 NSF Science and Technology Center for Engineering Mechanobiology, Washington University in St. Louis, St. Louis, 63130 MO USA

**Keywords:** Mechanotransduction, Mesenchymal stem cells, Biomaterials - cells, Biomaterials - cells

## Abstract

During mesenchymal development, the sources of mechanical forces transduced by cells transition over time from predominantly cell-cell interactions to predominantly cell-extracellular matrix (ECM) interactions. Transduction of the associated mechanical signals is critical for development, but how these signals converge to regulate human mesenchymal stem cells (hMSCs) mechanosensing is not fully understood, in part because time-evolving mechanical signals cannot readily be presented in vitro. Here, we established a DNA-driven cell culture platform that could be programmed to present the RGD peptide from fibronectin, mimicking cell-ECM interactions, and the HAVDI peptide from N-cadherin, mimicking cell-cell interactions, through DNA hybridization and toehold-mediated strand displacement reactions. The platform could be programmed to mimic the evolving cell-ECM and cell-cell interactions during mesenchymal development. We applied this platform to reveal that RGD/integrin ligation promoted cofilin phosphorylation, while HAVDI/N-cadherin ligation inhibited cofilin phosphorylation. Cofilin phosphorylation upregulated perinuclear apical actin fibers, which deformed the nucleus and thereby induced YAP nuclear localization in hMSCs, resulting in subsequent osteogenic differentiation. Our programmable culture platform is broadly applicable to the study of dynamic, integrated mechanobiological signals in development, healing, and tissue engineering.

## Abstract (web summary)

Human mesenchymal stem cells differentiate in response to mechanical adhesive interactions in their microenvironment. Here, the authors develop a culture platform that can dynamically mimic the cell-cell and cell-extracellular matrix interactions associated with development and mesenchymal differentiation in vivo.

## Introduction

Human mesenchymal stem cells (hMSCs) sense and respond to the stiffness^1–5^, geometry^6–8^, topography^9–11^, and physical dimensions^12,13^ of their extracellular matrix (ECM), and commit to a fate that is directed in part by their mechanical microenvironment. Studies uncovering the pathways by which this occurs have been enabled by advances in the synthesis of substrates that mimic cell-ECM interactions^14–16^. However, these substrates are historically static, and a new generation of the material platform is now emerging that replicates how the in vivo microenvironment evolves over time in development, disease, injury, and healing^17^. Such material platforms include poly-pyrrole arrays with reversible switchable hydrophobicity and hydrophilicity via electrochemical reduction and oxidation, providing dynamic control of attachment and detachment of MSCs^18^, and modulation of RGD oscillations by oscillating magnetic fields, to regulate adhesion and differentiation of stem cells^19^. Cell culture platforms capable of recapitulating native dynamic cell-ECM interactions represent an important frontier.

Our focus was thus a culture platform to provide cells with dynamically switchable cues that mimic both cell-ECM and cell-cell interactions. We were motivated by the finding that cadherin-based cell-cell mechanical interactions initiate downstream signaling that regulates cell fate^20–22^. Two primary pieces of evidence exist for this. First, sparse cells (with limited cell-cell interactions) exhibit more nuclear localization of Yes-associated protein (YAP) than dense cells (with widespread cell-cell interactions)^23^. YAP translocation to the nucleus regulates mechanotransduction and differentiation of MSCs^8,24^. Second, cadherin overexpression in developing limb buds promotes chondrogenesis, while cadherin inhibition attenuates chondrogenesis^25^. In MSCs, cell-cell adhesions are mediated by homotypic interactions of N-cadherin on adjacent cells, and the HAVDI motif in the first extracellular domain of N-cadherin acts to mimic this interaction^26^. Although these effects can be switched off gradually in a hyaluronic acid (HA) hydrogel with HAVDI containing a metalloprotease ADAM10-cleavable domain via cell-surface ADAM10^27^, on-demand, controlled switching of HAVDI is not yet available.

Cadherin-based (cell-cell) and integrin-based (cell-ECM) signals are antagonistic in their regulation of mechanotransduction, likely due to their separate linkages to the actin cytoskeleton through adaptive proteins, and to the overlap of signaling proteins such as Rho GTPase^28,29^. A key platform for quantifying this interplay is that of Cosgrove et al.^30^, who developed a HA hydrogel system with decoupled presentation of RGD and HAVDI peptides and used it to discover the competition between cell-cell and cell-ECM signaling that determines the nuclear localization of YAP. Parallel technologies mimicking cell-cell and cell-ECM interactions include micropatterned domains of collagen and cadherin on substrates^31^ and light-mediated thiol-norbornene chemistry to encapsulate cells within hydrogels that are patterned with HAVDI and RGD peptides^32^. However, cell-ECM and cell-cell cues vary over time during mesenchymal development, with the rich cell-cell signaling of early development gradually being replaced by cell-ECM signaling^30,33^. No currently available platform allows programmable variation of these cues over time to mimic this feature of mesenchymal development. We therefore developed a platform for doing so, and used it to study how time-evolving mechanical signals arise from cell-ECM and cell-cell interactions regulate the mechanosensing of hMSCs.

Our platform exploits the highly programmable nature of DNA to anchor signal molecules to a substrate^34–38^. RGD-DNA and HAVDI-DNA molecules were conjugated to poly(ethylene glycol) (PEG) hydrogels through DNA hybridization, and these peptide-DNA molecules could be removed from the PEG hydrogel by a toehold-mediated strand displacement reaction. This enabled nondestructive control of cell-ECM and cell-cell interactions through soluble, compatible molecules, without the need for factors that are potentially damaging to the cell or the hydrogel, such as photons^39^ or electrochemical potentials^18^. This platform enabled simultaneously programmable presentation of RGD and HAVDI to mimic the evolving cell-ECM and cell-cell interactions during mesenchymal development. We applied this system to reveal that RGD/integrin and HAVDI/N-cadherin ligations competitively regulate cofilin phosphorylation, thereby regulating downstream hMSCs mechanosensing.

## Results and discussion

### Tunable hydrogels presenting dynamic cell-ECM and cell-cell cues

**Fig. 1:**
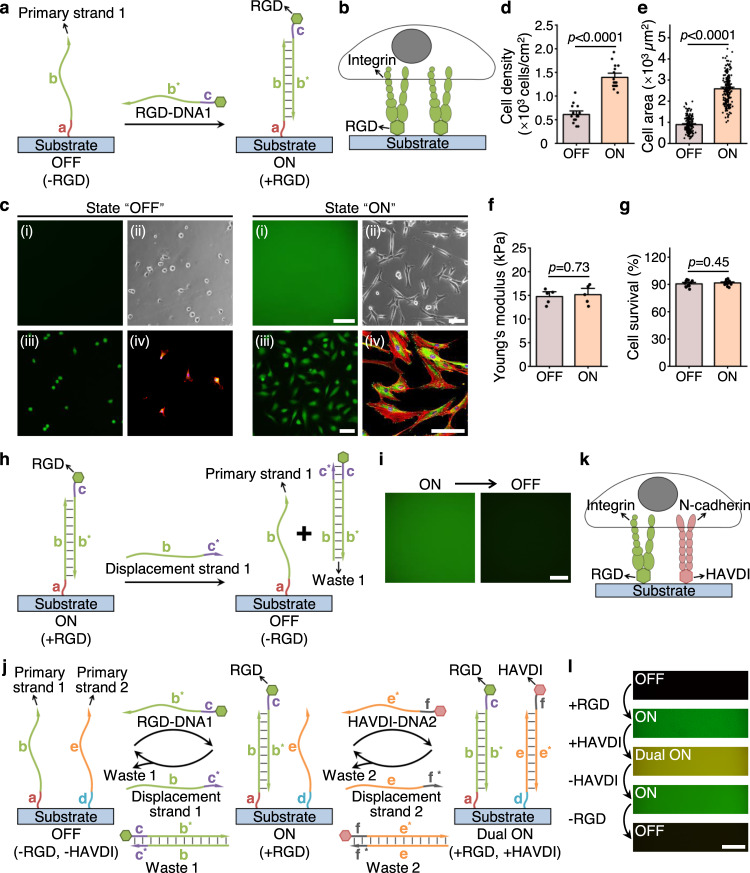
Programmable presentation of integrin and N-cadherin adhesive domains to study hMSCs mechanosensing. **a** Schematic of RGD conjugation to PEG hydrogels by DNA hybridization. DNA strands were drawn as lines with arrows at the 3′ end. “*” indicates complementary sequences. **b** “ON” substrate allows for integrin-RGD adhesive interactions. **c** Characterization of RGD conjugation and hMSCs adhesion on “OFF” and “ON” substrates, as shown by (i) fluorescence images of FAM-labeled RGD (this experiment was repeated independently 3 times with similar results), (ii) bright-field, (iii) live-dead staining (green for live cells and red for dead cells) and (iv) immunostaining for paxillin (green), F-actin (red), and nuclei (blue). **d** Cell density (from left to right *n* = 15, 17 samples) and **e** spreading area of hMSCs (from left to right *n* = 163, 194 cells) on “ON” and “OFF” substrates. **f** Young’s moduli of “ON” and “OFF” substrates (*n* = 5 samples per group). **g** Quantification of hMSCs viability on “ON” and “OFF” substrates (from left to right *n* = 11, 13 samples). **h** Schematic of RGD removal from “ON” hydrogels using a fully complementary displacement strand through a toehold-mediated strand displacement reaction. Toehold domain was labelled with lowercase “c”. **i** Fluorescence images of removal of FAM-labeled RGD from the “ON” substrates (this experiment was repeated independently 3 times with similar results). **j** Schematic of programmable presentation of RGD and HAVDI. A hydrogel was modified with two different primary strands to bind to RGD and HAVDI, respectively. RGD-DNA1 and HAVDI-DNA2 bearing orthogonal toeholds allowed selective removal of either signal via its complementary displacement strand. Toehold domains were labelled with lowercase letters “c” and “f”. **k** “Dual ON” substrates allow for RGD/integrin and HAVDI/N-cadherin ligations. **l** Fluorescent tagging allowed for visualization of peptide conjugation and removal on the PEG substrates (this experiment was repeated independently 3 times with similar results). Data are presented as mean ± s.e.m., and *p* values were obtained using one-way ANOVA followed by Tukey’s post hoc test (**d**, **e**, **f**, **g**). Scale bars: 100 µm (**c**, **i**, **l**). Source data are provided as a Source Data file.

A PEG hydrogel was generated that enables the programmable presentation of RGD and HAVDI (Supplementary Fig. 1). To promote cell adhesion, RGD-DNA1 molecule was conjugated via DNA hybridization to complementary DNA (referred to as “primary strand 1”) immobilized on a PEG hydrogel (Fig. 1a; Supplementary Fig. 2). In the “OFF” state, with no peptides presented on the hydrogel, hMSCs adhesion was inhibited, while in the “ON” state, with RGD conjugated to the hydrogel, hMSCs adhesion occurred by RGD/integrin ligation that mimicked cell-ECM interactions (Fig. 1b). Fluorescent tagging allowed for visualization of FAM-labeled RGD conjugation in the PEG hydrogel (Fig. 1ci). The “OFF” substrate had weak cell adhesion due to the presence of single-stranded DNA^40^. Therefore, although the PEG hydrogel itself would have no cell adhesion^39,41,42^, the “OFF” substrate served as a control group without RGD but with weak cell adhesion (Supplementary Fig. 3). The number and spreading area of attached hMSCs significantly increased on “ON” substrates compared with “OFF” substrates (Fig. 1cii, iv, d, e). The Young’s moduli of hydrogels with or without RGD were not significantly different (Fig. 1f), suggesting that the difference in spreading area on “ON” and “OFF” substrates was due to the presence or absence of RGD rather than to a change of stiffness. hMSCs viability was over 90% on both “ON” and “OFF” substrates after 5 days in culture (Fig. 1ciii, g), confirming their cytocompatibility.

A key advantage of our platform is that peptides can be both added and removed on the hydrogels. To remove peptides, we engineered a single-stranded overhang region (referred to as “toehold”) at the 5′ end of DNA1. Addition of displacement strand (referred to as “displacement strand 1”) triggered a rapid, toehold-mediated strand displacement reaction that released RGD from the hydrogel (Fig. 1h), as evident by the dissociation of FAM-labeled RGD from the substrates (Fig. 1i). State switching between the “OFF” and “ON” required 10-20 min (Supplementary Fig. 4).

We next demonstrated that the DNA-mediated platform enabled dynamic, independent switching of multiple signals by peptide-DNA molecules responsive to orthogonal triggers. As a proof of principle, we first constructed hydrogels conjugated with two different primary strands, one to bind RGD peptide from fibronectin (mimicking cell-ECM interactions) and another one to bind HAVDI peptide from N-cadherin (mimicking cell-cell interactions) using their respective peptide-DNA molecules (RGD-DNA1 and HAVDI-DNA2). Hydrogels functionalized with two primary strands were successively treated with FAM-labeled RGD-DNA1 and TAMRA-labeled HAVDI-DNA2, and then successively incubated in the displacement strand 2 and displacement strand 1 (Fig. 1j–l; Supplementary Fig. 5). Peptide-DNA strands were designed to be responsive to orthogonal toehold sequences, enabling dynamic regulation of cell-ECM and cell-cell interactions through on-demand conjugation or removal of RGD and HAVDI. Homogeneous distribution of peptides in PEG hydrogel was verified through fluorescent peptide tagging and fluorescence imaging (Supplementary Fig. 6a–c). No phase separation in the two-peptide distribution was observed in the PEG hydrogels (Supplementary Fig. 6d). HAVDI bound to the ventral side of cells adhered to RGD functionalized substrates (state “ON”), as was evident through live cell time-lapse videos of TAMRA-labeled HAVDI during state switching from “ON” to “Dual ON” (Supplementary Movie 1 and Supplementary Fig. 7). Conjugation and release of DNA-mediated peptides were highly sequence-specific (Supplementary Figs. 8, 9), even when multiple switches were present on the same substrate (Supplementary Fig. 10). State switching between the “ON” and “Dual ON” required 10-20 min (Supplementary Fig. 11).

Hydrogel stiffness could be tuned independently from 3–30 kPa by varying concentrations of PEG-SH with a constant concentration of PEG-MAL (Supplementary Fig. 12). Modification with RGD and HAVDI did not affect the stiffness of PEG hydrogels (Supplementary Fig. 12). To check the availability of RGD and HAVDI conjugated to the hydrogels, we evaluated the density and spacing of peptides on the hydrogel surface, which fell into a reasonable range (Supplementary Note 1). Additionally, hydrogels modified with HAVDI alone did not support cell adhesion and spreading (Supplementary Fig. 13). No significant difference in cell spreading area was observed between “ON” and “Dual ON” substrates (Supplementary Fig. 14). These findings support the notion that cell spreading in this system is driven by RGD and not altered by the additional presentation of HAVDI, in accordance with previous studies^30,43^.

### RGD/integrin and HAVDI/N-cadherin ligations competitively modulate hMSCs mechanosensing

**Fig. 2:**
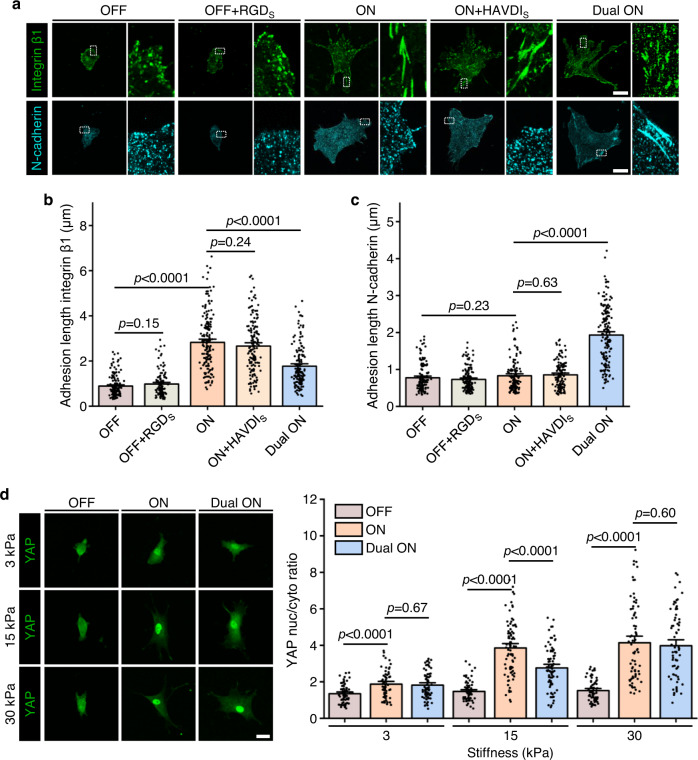
Integrin and N-cadherin clustering in hMSCs on various functionalized hydrogels and stiffness-dependent YAP nuclear localization associated with integrin and N-cadherin signaling. **a** Representative integrin β1 and N-cadherin images in hMSCs on various functionalized substrates (15 kPa) for 1 d. The subscript S indicated that RGD_S_ and HAVDI_S_ were the scrambled sequence of RGD and HAVDI, respectively. RGD ligation caused integrin clustering, and HAVDI ligation caused N-cadherin clustering. **b** Corresponding quantification of integrin β1 adhesion length from immunofluorescence images for the same conditions as **a** (from left to right *n* = 146, 122, 172, 151, 159 adhesions in 44, 38, 53, 45, 48 cells, respectively). **c** Corresponding quantification of N-cadherin adhesion length from immunofluorescence images for the same conditions as **a** (from left to right *n* = 143, 149, 144, 129, 166 adhesions in 41, 47, 45, 42, 51 cells respectively). **d** Left: Representative YAP images in hMSCs on 3, 15 and 30 kPa of “OFF”, “ON” or “Dual ON” substrates for 1 d. Right: Corresponding quantification of YAP nuc/cyto ratios (from left to right *n* = 64, 61, 72, 74, 85, 81, 68, 77, 71 cells). Data are presented as mean ± s.e.m., and *p* values were obtained using one-way ANOVA followed by Tukey’s post hoc test (**b**, **c**, **d**). Scale bars: 20 µm (**a**), 30 µm (**d**). Source data are provided as a Source Data file.

We next verified that RGD and HAVDI ligation activated integrin and N-cadherin in our system by checking whether, as in other systems, ligation enabled clustering^44,45^. The baseline levels of integrin and N-cadherin expression in hMSCs had negligible difference across batches of cells seeded on substrates with different ligand presentation (Supplementary Fig. 15). Substrates with RGD (state “ON”) induced integrin clustering, while substrates lacking RGD (state “OFF”) or with scramble RGD did not (Fig. 2a, b). Similarly, substrates with HAVDI (state “Dual ON”) induced N-cadherin clustering, while substrates lacking HAVDI or with scrambled HAVDI did not (Fig. 2a, c). Notably, HAVDI presentation (state “Dual ON”) decreased integrin clustering significantly (Fig. 2a, b), indicating that HAVDI/N-cadherin ligation partly suppressed integrin-driven mechanotransduction, in accordance with the previous studies^30^. To further confirm this finding, we verified it with two other types of integrin binding peptides (GFOGER and IKVAV), and obtained similar results (Supplementary Fig. 16). These observations suggest that RGD and HAVDI in our system provide cell signaling and activate canonical integrin and N-cadherin responses.

We then explored how RGD/integrin and HAVDI/N-cadherin ligations affected YAP signaling. Consistent with previous studies^46,47^, RGD presentation (“ON” substrates) significantly increased the YAP nuclear-to-cytoplasmic (nuc/cyto) ratios in hMSCs compared with “OFF” substrates at all stiffness (Fig. 2d). The YAP nuc/cyto ratios reduced with HAVDI presentation (“Dual ON” substrates) compared with “ON” substrates on intermediate stiffness substrates (15 kPa). However, HAVDI presentation did not alter YAP nuc/cyto ratios at either the upper or the lower bounds of substrate stiffness investigated (3 or 30 kPa), in accordance with previous studies^30,43^. To further understand these findings, we switched the state from “ON” to a partial “OFF” on 15 kPa substrates by adding varying amounts of the displacement strand 1. Partial release of RGD from “ON” substrates reduced YAP nuclear localization in a manner similar to reductions observed on “Dual ON” substrates (Supplementary Fig. 17). These observations suggest that HAVDI/N-cadherin ligation acts to offset the sensitivity of integrin-driven YAP signaling in hMSCs.

To reveal the mechanistic basis for these observations, we explored the expression of Lamin A, a mechanosensitive protein whose expression is highly correlated with tissue stiffness^48^. We quantified how Lamin A expression varied with ligand presentation over a range of stiffness using immunostaining and found a similar response to that of YAP (Supplementary Fig. 18), with RGD enhancing expression in hMSCs regardless of substrate stiffness, and HAVDI presentation decreasing expression only at intermediate substrate stiffness (15 kPa). Taken together, these results further highlight that HAVDI/N-cadherin ligation attenuates integrin-driven mechanosensing in hMSCs cultured on substrates of intermediate stiffness.

Next, a competition study was performed to determine if treating hMSCs with soluble waste 1 or 2 would abrogate the effects arising from the immobilized RGD or HAVDI on the substrates (15 kPa hydrogels were applied in the following study). Soluble waste 1 and 2 without the thiol group (referred to as “-SH”) were designed to bind with membrane integrin and N-cadherin receptors while avoiding conjugation to the PEG substrate. Blocking integrin with this soluble waste 1 (-SH) did not change the degree of YAP nuclear localization in hMSCs on “OFF” substrate, but significantly reduced YAP nuclear localization in hMSCs on “ON” and “Dual ON” substrates (Supplementary Fig. 19). Blocking N-cadherin with this soluble waste 2 (-SH) had no significant effect on YAP nuclear localization in hMSCs on either “OFF” or “ON” substrates, but completely abrogated the response to HAVDI presentation on “Dual ON” substrate (Supplementary Fig. 19). Taken together, these observations indicate that RGD and HAVDI peptides need to be tethered in order to elicit the response in YAP nuclear localization.

To check if forces generated from integrin or N-cadherin rupture the double-stranded DNA linkers, we designed DNA linkers with two different rupture forces (Supplementary Fig. 20a and Supplementary Note 2). DNA linker 1 (presented cells with RGD signals) with 56 pN rupture force allowed significant cell adhesion, while DNA linker 1 with 12 pN rupture force resulted in poor cell adhesion (Supplementary Fig. 20b). These observations suggest that the DNA linker 1 with 56 pN rupture force is stable enough to support integrin-mediated cell adhesion, in accordance with previous studies^35^. DNA linker 2 (presented cells with HAVDI signals) with both 12 and 56 pN rupture forces enabled delivering of HAVDI/N-cadherin signals as reflected by attenuation in YAP nuclear localization (Supplementary Fig. 20c). DNA linkers with 56 pN rupture force were thus used in all of the following studies. Next, we sought to determine how long the RGD and HAVDI signals by DNA linkers persisted. To this end, we measured the sizes of integrin and N-cadherin clusters that appeared in after 1 d or 14 d of ligation on “ON” or “Dual ON” substrates. We also quantified these for ligation of 1 d occurring after a waiting interval of 1 d or 14 d. No significant differences were observed, suggesting that the DNA linkers supply stable signals that persist for weeks (Supplementary Fig. 21).

**Fig. 3:**
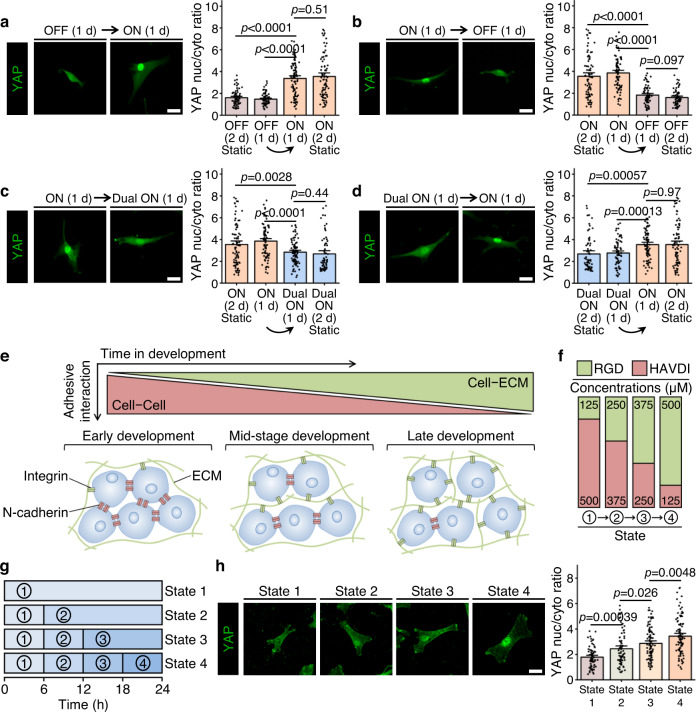
Programmable RGD/integrin and HAVDI/N-cadherin ligations regulate YAP signaling. **a** Left: Representative YAP images for state switching from “OFF” for initial 1 d to “ON” for subsequent 1 d. Right: Quantification of YAP nuc/cyto ratios (from left to right *n* = 67, 74, 93, 82 cells). Cells were cultured on continuously “OFF” and “ON” substrates for 2 d as control groups. **b** Left: Representative YAP images for state switching from “ON” for initial 1 d to “OFF” for subsequent 1 d. Right: Quantification of YAP nuc/cyto ratios (from left to right *n* = 82, 85, 71, 67 cells). Cells were cultured on continuously “ON” and “OFF” substrates for 2 d as control groups. **c** Left: Representative YAP images for state switching from “ON” for initial 1 d to “Dual ON” for subsequent 1 d. Right: Quantification of YAP nuc/cyto ratios (from left to right *n* = 82, 85, 96, 67 cells). Cells were cultured on continuously “ON” and “Dual ON” substrates for 2 d as control groups. **d** Left: Representative YAP images for state switching from “Dual ON” for initial 1 d to “ON” for subsequent 1 d. Right: Quantification of YAP nuc/cyto ratios (from left to right *n* = 67, 81, 79, 82 cells). Cells were cultured on continuously “Dual ON” and “ON” substrates for 2 d as control groups. **e** Schematic of the evolution of the mechanical microenvironment during mesenchymal development. **f** Schematic for achieving simultaneously increasing RGD and decreasing HAVDI on the substrates to mimic the mechanical microenvironment in **e**. **g** Concentrations of RGD and HAVDI were simultaneously varied every 6 h for the same conditions as **f**, until a total culture time of 24 h. **h** Left: Representative YAP images for the same conditions as **g**. Right: Quantification of YAP nuc/cyto ratios (from left to right *n* = 71, 79, 97, 94 cells). Data are presented as mean ± s.e.m., and *p* values were obtained using one-way ANOVA followed by Tukey’s post hoc test (**a**–**d**, **h**). Scale bars: 30 µm (**a–d**, **h**). Source data are provided as a Source Data file.

We set out to understand how the temporal presentation of RGD and HAVDI regulated hMSCs mechanosensing. Culturing cells on “ON” substrates for 2 d increased the YAP nuc/cyto ratios significantly compared to cells cultured on “OFF” substrates (Fig. 3a). State switching from “OFF” to “ON” at 1 d increased YAP nuc/cyto ratios (Fig. 3a), while state switching from “ON” to “OFF” at 1 d decreased YAP nuc/cyto ratios (Fig. 3b). Culturing cells on “Dual ON” substrates for 2 d markedly reduced the YAP nuc/cyto ratios compared to cells cultured on “ON” substrates (Fig. 3c). State switching from “ON” to “Dual ON” at 1 d induced a decrease in YAP nuc/cyto ratios, concomitant with a decrease in the integrin clustering and an increase in the N-cadherin clustering (Fig. 3c and Supplementary Fig. 22a–c). Conversely, state switching from “Dual ON” to “ON” at 1 d resulted in an increase in YAP nuc/cyto ratios, concomitant with an increase in the integrin clustering and a decrease in the N-cadherin clustering (Fig. 3d and Supplementary Fig. 22d–f). These observations suggest that integrin-mediated cell-ECM and N-cadherin-mediated cell-cell adhesive interactions in the system can be modulated dynamically.

We next tested whether hMSCs maintain mechanical memory in response to dynamic ligand switching (Supplementary Fig. 23a). hMSCs cultured on “ON” substrates for 1d, and then switched to “OFF” or “Dual ON” substrates for either 1 or 10 d, showed decreased YAP nuc/cyto ratios analogous to the levels found in cells that had never been exposed to “ON” substrate (“static control” group) (Supplementary Fig. 23b, c). This fully reversible YAP nuclear localization suggested that 1 d of exposure to “ON” substrate was a mechanical dosing that was inadequate for retention of mechanical memory, in accordance with previous studies^43,49,50^. However, 10 d culture on “ON” substrates and followed by switching to “OFF” substrates resulted in negligible reduction in YAP nuc/cyto ratios (Supplementary Fig. 23b, d), in accordance with prior observations^50,51^; while switching to “Dual ON” substrate resulted in partially reversible in YAP nuclear localization (Supplementary Fig. 23b, d), in accordance with previous studies^43^. Taken together, these data suggest that hMSCs integrate and store mechanical signals when exposed to “ON” substrates for 10 d, but that this mechanical memory could be partially erased by the HAVDI ligation that mimicked N-cadherin signaling in cell-cell interactions.

The cells that give rise to hMSCs trace their lineage back to the differentiation of mesenchyme from the mesoderm during gastrulation^52^. These cells exist in the absence of ECM proteins, and are in contact with neighboring cells. Over the course of further development, ECM proteins crowd out these cell-cell contacts^53^, and the extrinsic forces from these ECM proteins begin to shape subsequent hMSCs fate (Fig. 3e)^33^. That is, initial mechanical signaling switches progressively from mainly cell-cell interactions to mainly cell-ECM interactions as development progresses. To mimic the transition from cell-cell interactions to cell-ECM interactions during mesenchymal development, we first mimicked progressively increasing cell-ECM interactions by gradually increasing the amount of RGD on the substrate (Supplementary Fig. 24a–c). As the RGD concentrations increased, YAP nuc/cyto ratios increased, suggesting activated mechanotransduction of hMSCs (Supplementary Fig. 24d). To mimic the loss of cell-cell interactions, we gradually decreased the amount of HAVDI on the substrate while holding the concentration of RGD constant at 500 μM and again observed an increase in YAP ratios, concomitant with an increase in the integrin clustering and a decrease in the N-cadherin clustering (Supplementary Figs. 25, 26). To mimic the simultaneous changes of cell-ECM and cell-cell interactions associated with mesenchymal development, we held the total concentration of RGD and HAVDI peptides constant while changing their proportions (Fig. 3f, g) and found that as the RGD proportion increased, YAP nuclear localization increased (Fig. 3h), concomitant with an increase in the cell area and nuclear area (Supplementary Fig. 27). These results suggest that hMSCs mechanosensing is activated by the evolving mechanical microenvironment during mesenchymal development, and demonstrated that cell-ECM and cell-cell cues can be regulated individually or synergistically in our platform to orchestrate hMSCs mechanotransduction.

We  next studied how evolving cell-ECM and cell-cell interactions affects hMSCs differentiation by monitoring nuclear localization of Runt-related transcription factor 2 (RUNX2). RUNX2, a transcriptional partner of YAP that can activate alkaline phosphatase (ALP) expression and regulate osteogenesis^54,55^, serves as a marker of osteogenic differentiation of hMSCs. Similar to YAP ratios, the RUNX2 nuc/cyto ratios increased with RGD presentation (state “ON”), while decreased with HAVDI incorporation (state “Dual ON”) (Supplementary Fig. 28a), and scaled monotonically with variations in the proportion of RGD when the total concentration of RGD and HAVDI peptides was kept constant (Supplementary Fig. 28b–d). These data indicate progressive osteogenic commitment, and suggest temporal regulation of RGD/integrin and HAVDI/N-cadherin ligations can be used to affect hMSCs fate.

### RGD/integrin and HAVDI/N-cadherin ligations competitively regulate contractility of hMSCs

**Fig. 4:**
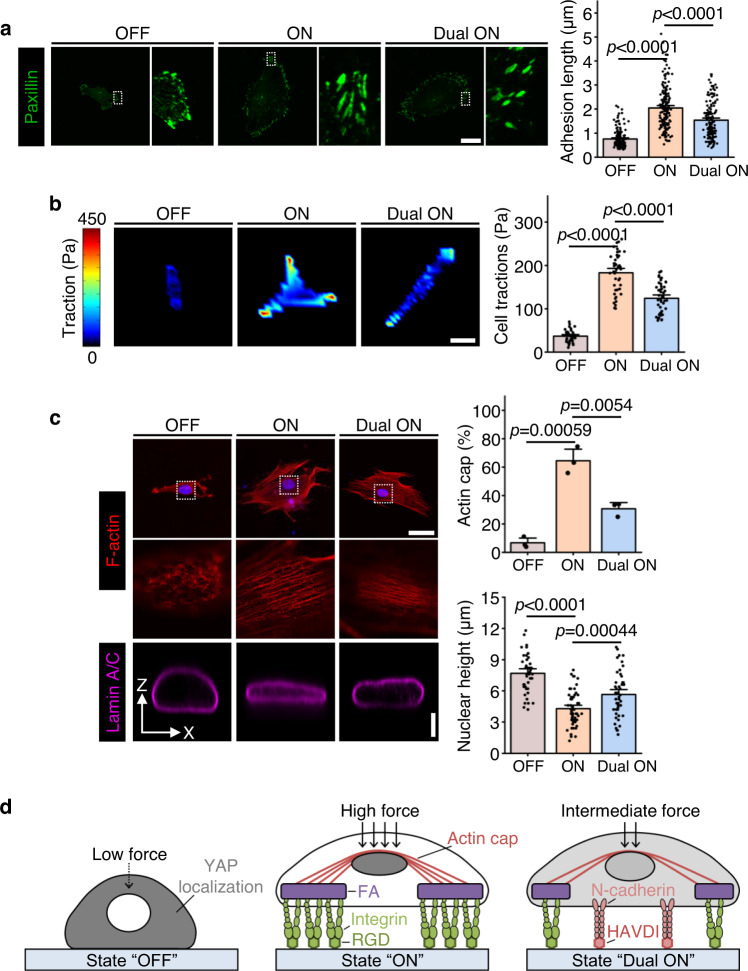
RGD/integrin and HAVDI/N-cadherin ligations regulate the adhesive state and the cytoskeletal organization of hMSCs. **a** Left: Representative paxillin images in hMSCs on “OFF”, “ON” and “Dual ON” substrates for 1 d. Zoomed regions correspond to the rectangles marked in white in the main images. Right: Corresponding quantification of paxillin adhesion length (from left to right *n* = 146, 173, 154 adhesions in 41, 47, 45 cells respectively). **b** Left: Representative heat maps of traction stress in hMSCs on “OFF”, “ON” and “Dual ON” substrates for 1 d. Right: Corresponding quantification of average traction stress per cell (from left to right *n* = 37, 44, 47 cells). **c** Left: Representative F-actin images in hMSCs on “OFF”, “ON” and “Dual ON” substrates for 1 d (top). Zoomed regions display details of F-actin organization in the apical region of the nucleus (middle). The cross-sectional side view of lamin A/C staining was captured along the XZ-plane crossing the center of the nucleus (bottom). Right: Corresponding quantification of the percentages of hMSCs having the actin cap (top) (*n* = 3 experiments per group). Corresponding quantification of nuclear height (bottom) (from left to right *n* = 43, 55, 48 cells). **d** Schematic of a possible pathway for YAP nuclear translocation in response to the RGD/integrin and HAVDI/N-cadherin ligations. RGD on “ON” substrates induced integrin clustering and promoted formation of focal adhesions (FA) and actin cap. Contractile force from actin cap compressed and flattened the nucleus, and may have thereby enabled nuclear localization of YAP. HAVDI on “Dual ON” substrates reduced integrin clustering and FA formation, possibly attenuating nuclear compression and nuclear localization of YAP. Data are presented as mean ± s.e.m., and *p* values were obtained using one-way ANOVA followed by Tukey’s post hoc test (**a**–**c**). Scale bars: 20 µm (**a**), 30 µm (**b**), 30 µm for F-actin images and 5 µm for lamin A/C images (**c**). Source data are provided as a Source Data file.

We next set out to determine if RGD/integrin and HAVDI/N-cadherin ligations alter downstream mechanosensitive signaling through alterations in the contractile state of hMSCs. To accomplish this, we examined the responses of the adhesive states to RGD/integrin and HAVDI/N-cadherin ligations. Paxillin immunostaining was performed to visualize focal adhesions (FA) in hMSCs. Results showed that hMSCs seeded on “ON” substrates had significantly longer FA than those seeded on “OFF” substrates, and that hMSCs cultured on “Dual ON” substrates had FA of intermediate length (Fig. 4a). Cell contractility scaled with FA length, as evident from traction force microscopy measurements (Fig. 4b), suggesting that RGD/integrin and HAVDI/N-cadherin ligations can ‘compete’ to alter hMSCs contractility.

We next affirmed how RGD/integrin and HAVDI/N-cadherin ligations affect the perinuclear apical actin cables (*i.e*. actin cap)^56–58^ and the degree to which the nucleus is compressed by the actin cytoskeleton. Results showed that the fraction of cells that formed an actin cap was smallest on “OFF” substrates, largest on “ON” substrates, and intermediate on “Dual ON” substrates (Fig. 4c). Lamin A/C immunostaining performed to investigate the response of the cell nucleus to these mechanical stimuli showed that nuclear height was lowest on “ON” substrates, highest on “OFF” substrates, and intermediate on “Dual ON” substrates, while nuclear area presented the opposite trend (Fig. 4c; Supplementary Fig. 29). No significant difference in nuclear volume was observed on these substrates (Supplementary Fig. 29). These results affirm that RGD/integrin and HAVDI/N-cadherin ligations regulate the actin cap formation and nuclear flattening, shown previously to drive YAP nuclear translocation (summarized in Fig. 4d)^43^.

**Fig. 5:**
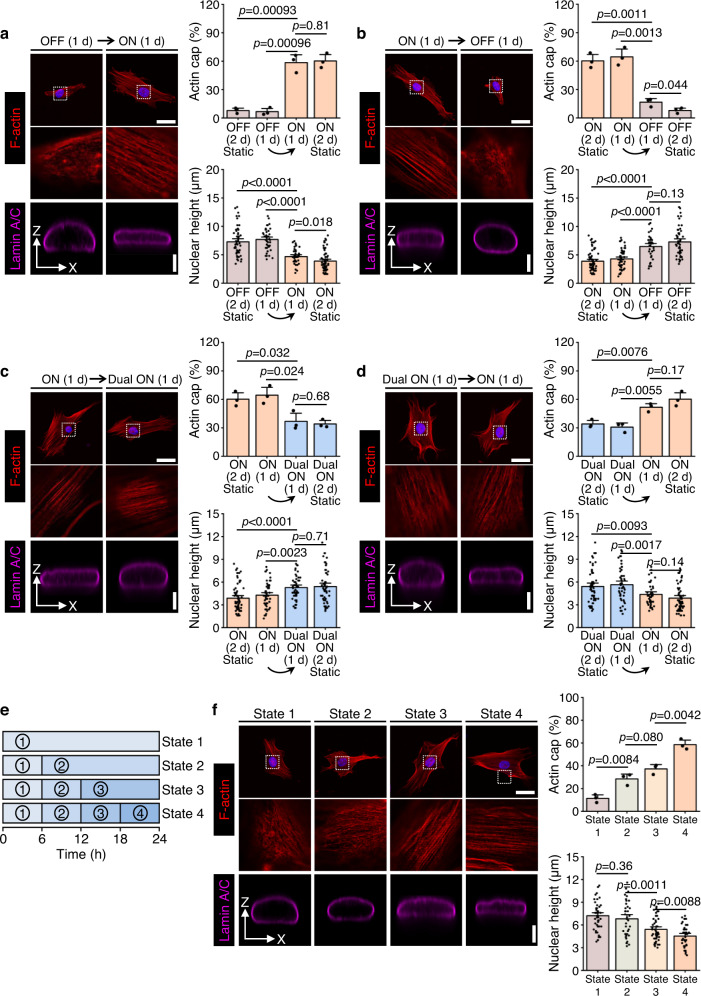
Programmable RGD/integrin and HAVDI/N-cadherin ligations regulate actin cap formation and nuclear height. **a** Left: Representative F-actin and lamin A/C images for state switching from “OFF” for initial 1 d to “ON” for subsequent 1 d. Right: Quantification of the percentages of hMSCs having the actin cap (*n* = 3 experiments per group) and nuclear height (from left to right *n* = 51, 43, 39, 63 cells). **b** Left: Representative F-actin and lamin A/C images for state switching from “ON” for initial 1 d to “OFF” for subsequent 1 d. Right: Quantification of the percentages of hMSCs having the actin cap (*n* = 3 experiments per group) and nuclear height (from left to right *n* = 63, 55, 35, 51 cells). **c** Left: Representative F-actin and lamin A/C images for state switching from “ON” for initial 1 d to “Dual ON” for subsequent 1 d. Right: Quantification of the percentages of hMSCs having the actin cap (*n* = 3 experiments per group) and nuclear height (from left to right *n* = 63, 55, 46, 55 cells). **d** Left: Representative F-actin and lamin A/C images for state switching from “Dual ON” for initial 1 d to “ON” for subsequent 1 d. Right: Quantification of the percentages of hMSCs having the actin cap (*n* = 3 experiments per group) and nuclear height (from left to right *n* = 55, 48, 42, 63 cells). **e** Concentrations of RGD and HAVDI were simultaneously varied every 6 h for the same conditions as Fig. 3f. **f** Left: Representative F-actin and lamin A/C images in hMSCs for the same conditions as **e**. Right: Corresponding quantification of the percentages of hMSCs having the actin cap (*n* = 3 experiments per group) and nuclear height (from left to right *n* = 46, 38, 51, 37 cells). Data are presented as mean ± s.e.m., and *p* values were obtained using one-way ANOVA followed by Tukey’s post hoc test (**a**–**d**, **f**). Scale bars: 30 µm for F-actin images and 5 µm for lamin A/C images (**a**–**d**, **f**). Source data are provided as a Source Data file.

We next sought to determine how the dynamics of RGD/integrin and HAVDI/N-cadherin ligations affect the mechanical state of hMSCs. State switching from “OFF” to “ON” at 1 d promoted the formation of actin cap in hMSCs, accompanied by a reduction of nuclear height (Fig. 5a). Conversely, the actin cap vanished when state switching from “ON” to “OFF” at 1 d, and the nuclear height was restored (Fig. 5b). State switching from “ON” to “Dual ON” at 1 d decreased actin cap formation and increased nuclear height to intermediate levels (Fig. 5c), while state switching from “Dual ON” to “ON” reversed these trends (Fig. 5d). Applying the system to mimic the evolution from HAVDI/N-cadherin ligation to RGD/integrin ligation in the mechanical microenvironment associated with mesenchymal development showed that actin cap formation concomitant with a decrease in nuclear height, and that both scaled with the fraction of RGD ligands on the substrates (Fig. 5e, f). These findings indicate that temporal presentation of RGD/integrin and HAVDI/N-cadherin ligations dynamically regulates actin cap formation and nuclear height.

### RGD/integrin and HAVDI/N-cadherin ligations alter hMSCs mechanosensing via competitively regulating cofilin phosphorylation

**Fig. 6:**
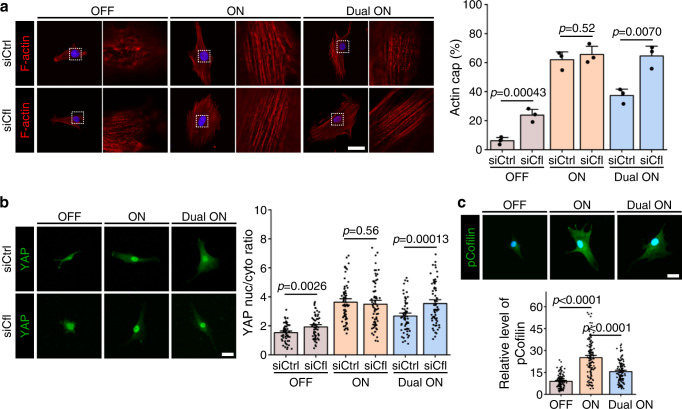
RGD/integrin and HAVDI/N-cadherin ligations affect hMSCs mechanosensing by regulating cofilin phosphorylation. **a** Left: Representative F-actin images in control and cofilin-depleted hMSCs on “OFF”, “ON” and “Dual ON” substrates for 1 d. Zoomed regions display details of F-actin organization in the apical region of the nucleus. Right: Corresponding quantification of the percentages of hMSCs having an actin cap (*n* = 3 experiments per group). **b** Left: Representative YAP images in control and cofilin-depleted hMSCs on “OFF”, “ON” and “Dual ON” substrates for 1 d. Right: Corresponding quantification of YAP nuc/cyto ratios (from left to right *n* = 62, 58, 68, 72, 65, 68 cells). **c** Top: Representative pCofilin images in hMSCs on “OFF”, “ON” and “Dual ON” substrates for 1 d. Bottom: Corresponding quantification of pCofilin level based on the immunostaining (from left to right *n* = 107, 136, 118 cells). Data are presented as mean ± s.e.m., and *p* values were obtained using one-way ANOVA followed by Tukey’s post hoc test (**a**–**c**). Scale bars: 30 µm (**a**–**c**). Source data are provided as a Source Data file.

On the basis of the findings above, we next sought to determine the mechanism by which RGD/integrin and HAVDI/N-cadherin ligations alter the mechanical state of hMSCs. Integrin and N-cadherin serve as signalling hubs for Rac1^30,59,60^. Actin-bundling proteins downstream of Rac1, such as cofilin, can control YAP localization^23^. Cofilin is known to be a regulator of actin filament dynamics, and its ability to bind and depolymerize actin is abolished by phosphorylation at Ser 3^61,62^. We tested the hypothesis that cofilin plays a role in hMSCs mechanosensing in response to RGD/integrin and HAVDI/N-cadherin ligations. To accomplish this, we knocked down cofilin expression in hMSCs using siRNA (Supplementary Fig. 30). In control hMSCs treated with inactive siRNA, the actin cap percentages and YAP nuc/cyto ratios were smallest on “OFF” substrates, largest on “ON” substrates, and intermediate on “Dual ON” substrates (Fig. 6a, b). Cofilin depletion upregulated these actin cap percentages and YAP nuc/cyto ratios in hMSCs cultured on “OFF” and “Dual ON” substrates, but not on “ON” substrates (Fig. 6a, b). Notably, cofilin depletion restored the actin cap percentages and YAP nuc/cyto ratios in hMSCs cultured on “Dual ON” substrates to the levels observed in hMSCs cultured on “ON” substrates (Fig. 6a, b). Cofilin depletion promoted paxillin clustering, a proxy for focal adhesion formation, in hMSCs on “OFF” and “Dual ON” substrates (Supplementary Fig. 31). These observations implicate cofilin as a key mediator of hMSCs mechanosensing.

We then sought to understand why cofilin depletion did not affect actin cap formation and YAP nuclear localization on “ON” substrates. To this end, we performed cofilin knock-down experiment on “ON” substrates of different stiffness (3, 15, 30 kPa). On softer “ON” substrates (3 kPa), cofilin depletion promoted the formation of actin cap and nuclear YAP localization, while on stiffer (15 or 30 kPa) substrates, cofilin depletion had negligible effects (Supplementary Fig. 32). These observations are in accordance with a previous study showing cofilin, as an F-actin-severing protein, to be a gatekeeper limiting YAP activity in cells experiencing low mechanical stresses, including contact inhibition of proliferation^23^. Taken together, these findings suggest that cofilin inhibits actin cap formation and YAP nuclear localization only when hMSCs are in a low contractile state.

We also explored a potential role of profilin, an actin-binding protein whose phosphorylation at Tyr 129 promotes binding to actin and actin polymerization^63,64^. To this end, we seeded hMSCs on “OFF”, “ON” and “Dual ON” substrates, then quantified phosphorylation levels of profilin. Results showed that RGD/integrin ligation promoted phosphorylation of profilin, but that HAVDI/N-cadherin ligation had negligible effect, suggesting a potential role of actin polymerization in the phenomena observed (Supplementary Fig. 33).

To explicitly confirm that the relationship between actin cap formation and RGD/integrin or HAVDI/N-cadherin ligations was governed by cofilin phosphorylation, we performed phosphorylated cofilin (pCofilin) immunostaining in hMSCs on “OFF”, “ON” and “Dual ON” substrates. hMSCs seeded on “ON” substrates showed higher pCofilin levels than those seeded on “OFF” substrates, and HAVDI presentation (“Dual ON” substrates) reduced pCofilin levels (Fig. 6c). This conclusion was verified by additional quantification performed using ELISA (Supplementary Fig. 34). pCofilin was found primarily in the nuclei of cells on “OFF” substrates (Supplementary Fig. 35). RGD presentation (“ON” substrates) induced partial translocation of pCofilin to the cytoplasm, while HAVDI presentation inhibited this translocation (Supplementary Fig. 35). These observations are in accordance with previous reports that non-phosphorylated cofilin accumulates within nuclei, and that dephosphorylation of Ser 3 regulates the nuclear translocation of cofilin^65^. These findings suggest that RGD/integrin and HAVDI/N-cadherin ligations antagonistically regulate cofilin phosphorylation, with RGD/integrin ligation upregulating it, and HAVDI/N-cadherin ligation downregulating it.

**Fig. 7:**
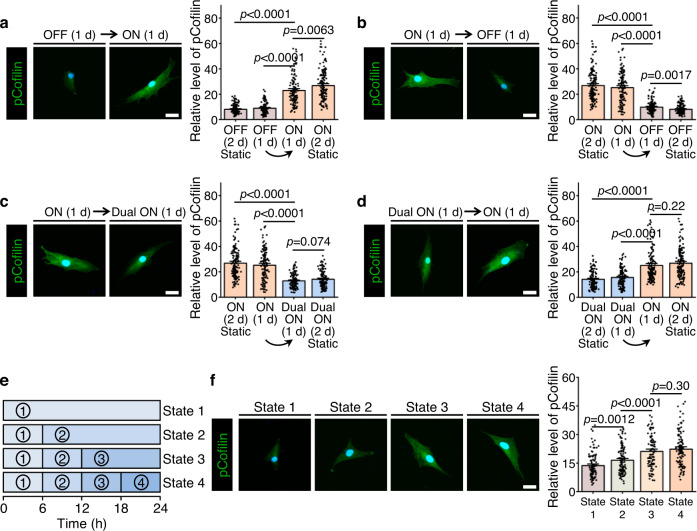
Programmable RGD/integrin and HAVDI/N-cadherin ligations regulate hMSCs mechanosensing by mediating cofilin phosphorylation. **a** Left: Representative pCofilin images in hMSCs for state switching from “OFF” for initial 1 d to “ON” for subsequent 1 d. Right: Corresponding quantification of pCofilin levels based on the immunostaining (from left to right *n* = 122, 107, 131, 144 cells). Cells were cultured on continuously “OFF” and “ON” substrates for 2 d as control groups. **b** Left: Representative pCofilin images for state switching from “ON” for initial 1 d to “OFF” for subsequent 1 d. Right: Corresponding quantification of pCofilin levels based on the immunostaining (from left to right *n* = 144, 136, 112, 122 cells). Cells were cultured on continuously “OFF” and “ON” substrates for 2 d as control groups. **c** Left: Representative pCofilin images for state switching from “ON” for initial 1 d to “Dual ON” for subsequent 1 d. Right: Corresponding quantification of pCofilin levels based on the immunostaining (from left to right *n* = 144, 136, 134, 127 cells). Cells were cultured on continuously “ON” and “Dual ON” substrates for 2 d as control groups. **d** Left: Representative pCofilin images for state switching from “Dual ON” for initial 1 d to “ON” for subsequent 1 d. Right: Corresponding quantification of pCofilin levels based on the immunostaining (from left to right *n* = 127, 118, 148, 144 cells). Cells were cultured on continuously “Dual ON” and “ON” substrates for 2 d as control groups. **e** Concentrations of RGD and HAVDI were simultaneously varied every 6 h for the same conditions as Fig. 3f, until a total culture time of 24 h. **f** Left: Representative pCofilin images for the same conditions as **e**. Right: Corresponding quantification of pCofilin levels based on the immunostaining (from left to right *n* = 109, 126, 103, 114 cells). Data are presented as mean ± s.e.m., and *p* values were obtained using one-way ANOVA followed by Tukey’s post hoc test (**a**–**d**, **f**). Scale bars: 30 µm (**a**–**d**, **f**). Source data are provided as a Source Data file.

To determine if the aforementioned mechanism is operative in the dynamic RGD/integrin and HAVDI/N-cadherin ligations, we quantified the levels of pCofilin in hMSCs during switching of substrate states. State switching from “OFF” to “ON” at 1 d increased pCofilin levels, while state switching from “ON” to “OFF” at 1 d reversed the trend (Fig. 7a, b; Supplementary Fig. 36a, b). State switching from “ON” to “Dual ON” at 1 d reduced pCofilin levels, and state switching from “Dual ON” to “ON” restored them (Fig. 7c, d and Supplementary Fig. 36c, d). Levels of pCofilin increased with increasing  proportion of RGD/integrin ligation on substrates with a time-evolving mechanical microenvironment mimicking mesenchymal development (Fig. 7e, f and Supplementary Fig. 36e, f). These observations indicate that temporal presentation of RGD/integrin and HAVDI/N-cadherin ligations modulate hMSCs mechanosensing by dynamically mediating phosphorylation and dephosphorylation of cofilin.

hMSCs in vivo receive mechanical cues from both cell-ECM and cell-cell interactions, with the former promoting mechanosensing and the latter inhibiting it. We showed that these two antagonistic factors affect YAP nuclear localization, and found that N-cadherin signaling can offset these effects arising from cell-ECM interactions. Our platform enabled on-demand, and programmable control of mechanobiological signals through DNA hybridization and toehold-mediated strand displacement reactions, thereby mimicking the transition from cell-cell to cell-ECM interactions during mesenchymal development. We applied this platform to reveal that RGD/integrin and HAVDI/N-cadherin ligations competitively regulate cofilin phosphorylation and subsequent changes in downstream mechanosensitive signaling. We believe that the dynamic control of substrate ligands offered by our platform will be useful for cell culture systems, and for clarifying how multiple extracellular mechanical cues regulate cell function in time-evolving manner.

It should be noted that mesenchymal development occurs over the course of days to months, and in a complex microenvironment with soluble factors such as cytokines and growth factors; with interactions such as those associated with nectin and Notch/Delta; and with multiple extracellular sources of mechanical stress^53^. Relative to the true mesenchymal niche during development, our experiments were brief and highly simplified. Importantly, the study enabled the identification of a pure mechanobiological pathway that affected hMSCs mechanosensing in response to time-evolving mechanical signals arising from integrin-mediated cell-ECM interactions and N-cadherin-mediated cell-cell interactions. Future investigations are needed to identify additional specific proteins and/or signalling pathways that regulate hMSCs mechanosensing.

## Methods

### Synthesis of peptide-DNA molecules

RGD and HAVDI were covalently conjugated to DNA1 and DNA2, respectively, via copper-free click chemistry reaction between the cyclooctyne on these peptides and the azide on the DNA. Briefly, equal molar ratio of azide-modified DNA and DBCO-modified peptides were dissolved in phosphate-buffered saline (PBS) for 24 h at room temperature to generate the peptide-DNA molecules. All oligonucleotides (Sangon Biotech) were listed in Supplementary Table 1. All peptides (Top-peptide Bio) were listed in Supplementary Table 2.

### Preparation of peptide-DNA-modified PEG hydrogel

PEG hydrogels were prepared by mixing 8-arm PEG maleimide (PEG-MAL, 10 kDa, JenKem Technology) and 8-arm PEG thiol (PEG-SH, 10 kDa, JenKem Technology) for 30 min at room temperature. To remove unreacted molecules, the hydrogels were rinsed thrice with PBS following each step. PEG hydrogels were treated with primary strand solution for 1 h for primary strand conjugation. Both of primary strand 1 and 2 were used at a final concentration of 500 μM in hydrogels. Then the primary strand-functionalized hydrogels were incubated with peptide-DNA solution for 1 h at room temperature to allow hybridization. FAM-labeled RGD and TAMRA-labeled HAVDI were used to characterize the conjugation of RGD or HAVDI in the hydrogels.

### Mechanical properties of hydrogels

A DHR3 shear rheometer (TA Instruments) with a parallel plate geometry (8 mm in diameter) was used for rheological testing. The storage modulus G′ and loss modulus G′′ were measured at 1% strain and a frequency of 1 rad s^−1^. The 14 mm diameter PEG hydrogel samples were tested at a constant temperature of 37 °C. Young’s modulus *E* was calculated as follows:

1 $$E=2\left(1+\nu \right)\sqrt {{G}^{\prime 2}+G^{{\prime 2}}}$$

where *ν* = 0.5 for the Poisson ratio of PEG hydrogels^51,66,67^.

### hMSCs isolation and culture

Human mesenchymal stem cells (hMSCs) were isolated from human bone marrow provided by commercial sources (Cyagen Biosciences). Briefly, cells were obtained from donors by bone marrow aspiration, then monocyte density centrifugation was performed and selected for adherent culture. Standard analytical methods were used to screen cell growth and differentiation into fat and bone. The cells from five donors were mixed in reserve before performing all the experiments in this manuscript. The age and sex of the five hMSCs donors were 36 years (male), 38 years (female), 38 years (female), 41 years (female), and 42 years (male), respectively. hMSCs were cultured in growth media (Cyagen, HUXMA-90011), except as noted. For osteogenic differentiation studies, cells were cultured in an osteogenic medium (Cyagen, HUXMA-90021) to assay the hMSCs osteogenic differentiation capability.

### Quantitative real-time PCR and ELISA

For real-time PCR, the total RNA was harvested using RNA extraction kit (Takara, 9767) according to the manufacturer’s instructions. A high-capacity RevertAid First Strand cDNA Synthesis Kit (Thermo Fisher Scientific; K1622) was used to transcribe the extracted RNA into cDNA by polymerase chain reaction (PCR). Quantitative real-time PCR (qRT-PCR) was conducted using SYBR® Premix Ex Taq™ II (Takara, RR820A) on a 7500 Fast Real-Time PCR System (Applied Biosystems). The relative mRNA expression is calculated relative to GAPDH. Sequences of primers were: GAPDH: fwd GCAAGAGCACAAGAGGAAGAG, rev AAGGGGTCTACATGGCAACT; Cofilin: fwd ATAAGGACTGCCGCTATGCC, rev ACCTCCTCGTAGCAGTTTGC. For enzyme-linked immunosorbent assay (ELISA), cells on hydrogels were placed on ice, rinsed twice with ice-cold PBS and scraped with RIPA buffer supplemented with protease and phosphatase inhibitors. Supernatant was harvested by centrifugation at 4 °C and 13,400 g for 10 min. Protein levels were analyzed by a commercial ELISA kit according to the manufacturer’s instructions. The ELISA kit used to quantify corresponding protein in this study contained pCofilin (Mlbio, YJ151871), cofilin (Mlbio, YJ369400), integrin β1 (Mlbio, YJ063099), N-cadherin (Mlbio, YJ027670), pProfilin (Mlbio, YJ591470), profilin (Mlbio, YJ560031), GAPDH (Mlbio, YJ038337).

### Dynamic regulation of RGD and HAVDI on the hydrogels

Hydrogels for cell culture were sterilized in 75% (v/v) aqueous ethanol for 3 h followed by five rinses with sterilized PBS. Prior to cell seeding, the hydrogels were prewetted with growth media for 30 min. hMSCs were plated at low density (2000 cells per cm^2^) to prevent cell-cell interactions. For RGD/integrin ligation, the RGD-DNA1 was added to “OFF” substrates to switch it to “ON” substrates. To switch back to the “OFF” substrates, the displacement strand 1 was added into “ON” substrates. For HAVDI/N-cadherin ligation, the HAVDI-DNA2 was added to “ON” substrates to switch it to “Dual ON” substrates. To switch back to the “ON” substrates, the displacement strand 2 was added into “Dual ON” substrate.

### Cell viability and transfections

For cell viability experiments, media was exchanged with PBS containing 2 μM Calcein-AM and 4 μM Ethidium homodimer-1 for 10 min at 37 °C. The cells were then rinsed with PBS and imaged with Olympus FV3000 confocal microscope. For downregulation of cofilin, the cells were transfected with corresponding siRNAs at a 100 nM concentration. The transfections were conducted using Opti-MEM Reduced Serum Medium (Thermo Fisher Scientific, 31985070) and Lipofectamine RNAiMAX reagent (Thermo Fisher Scientific, 13778030) according to the manufacturer’s instructions. The siRNAs used were cofilin siRNA I (Cell Signaling, 6267) and control siRNA (Cell Signaling, 6568).

### Immunostaining and quantification

Collected samples were fixed at desired time points using 4% paraformaldehyde for 20 min at room temperature. Samples were rinsed thrice with PBS, then permeabilized with 0.5% Triton X-100 for 10 min. Non-specific binding sites were subsequently blocked with 5% bovine serum albumin (BSA) for 30 min at room temperature. Samples were then incubated with primary antibody diluted in 1% BSA overnight at 4 °C. Primary antibodies and dilutions used in this study contained anti-Integrin β1 (1:500, mouse, Abcam, ab30394), anti-N-Cadherin (1:200, rabbit, Cell Signaling, 13116), anti-YAP (1:100, rabbit, Cell Signaling, 14074), anti-RUNX2 (1:1000, rabbit, Cell Signaling, 12556), anti-Paxillin (1:200, rabbit, Abcam, ab32084), anti-Lamin A/C (1:100, mouse, Cell Signaling, 4777), anti-Phospho-Cofilin (1:100, rabbit, Cell Signaling, 3313), anti-Lamin A (1:100, mouse, Cell Signaling, 86846). After three PBS rinses, AlexaFluor-488[H+L] secondary antibodies (1:500, goat anti-rabbit, Cell Signaling, 4412) and AlexaFluor-647[H+L] secondary antibodies (1:500, goat anti-mouse, Cell Signaling, 4410) were added for 2 h at room temperature, followed by F-actin staining using Rhodamine Phalloidin (1:1,000; Invitrogen, R415) incubated for 30 min. All immunostained samples were embedded in ProLong® Gold Antifade Reagent with DAPI (Cell Signaling, 8961) and visualized with Olympus FV3000 confocal microscope.

### Quantification of YAP and RUNX2 nuc/cyto ratio

For the YAP and RUNX2 nucleus-to-cytoplasm ratios, the nucleus and cytoplasm were identified by F-actin and DAPI staining, respectively, and the nuc/cyto ratio *R* for YAP or RUNX2 was calculated following a procedure used by others^51,68–70^, in which the ratio of the total fluorescence intensity in the nucleus, ${I}_{{nucleus}}$, to the total fluorescence in the remainder of the cell, was weighted by the areas of the nucleus and the remainder of the cell:

2 $$R=\frac{({I}_{{nucleus}}/{A}_{{nucleus}})\,}{({I}_{{cell}}-{I}_{{nucleus}})/({A}_{{cell}}-{A}_{{nucleus}})}$$

where ${A}_{{nucleus}}$ is the area of the nucleus as measured by DAPI staining, ${A}_{{cell}}$ is the overall area of the cell as delineated by F-actin staining, and ${I}_{{cell}}$ is the total fluorescence intensity in the overall cell. Intensities and areas were measured using Image J.

### Quantification of the actin cap percentage

To detect the presence or absence of an actin cap in each cell, actin anisotropy on the apical plane of the cell was quantified using a freely-available plugin for ImageJ, FibrilTool, described in detail in reference^71^. Briefly, confocal slices of the apical planes of the cell were taken, and the inner region of the cell was selected as the quantification area. Anisotropy ratios are indicated on a scale from 0 (perfectly isotropic) to 1 (perfectly anisotropic). We defined the threshold for the presence of an actin cap as being when the anisotropy ratios of actin on the apical plane of hMSCs exceeded or equaled to 0.2; anisotropy ratios below 0.2 indicated the absence of an actin cap.

### Traction force microscopy

8-arm PEG maleimide was doped with 0.2 μm diameter fluorescent microspheres at 2% v/v (Invitrogen, F8811), and then mixed with 8-arm PEG thiol to form hydrogels. hMSCs were cultured on the PEG hydrogels for 24 h before traction force microscopy (TFM) analysis was performed. Phase contrast images of cells and fluorescence images of the embedded nanobeads were captured on an Olympus FV3000 confocal microscope. Image sequences of each cell, taken before and after lysis of the cells with SDS (sodium dodecyl sulfate) buffer, were analyzed by a previously published MATLAB script^72^ to obtain a traction force map and the average traction stress exerted by cells on the underlying substrate.

### Statistical analysis

Statistical comparisons were performed with Origin (2020) using one-way analysis of variance (ANOVA) with Tukey’s post hoc test for comparison of multiple groups. The threshold for statistically significant differences between groups was *p* < 0.05. In all figures, data are shown as mean ± standard error of the mean (s.e.m.) unless otherwise stated. All experiments were repeated independently at least thrice, except where noted. The number of cells counted for each condition was indicated in each figure legend.

### Reporting summary

Further information on research design is available in the Nature Research Reporting Summary linked to this article.

## Supplementary information

Supplementary Information

Description of Additional Supplementary Files

Supplementary Movie 1

Reporting Summary

## Data Availability

All data in this study are available in the manuscript and the Supplementary materials or from the corresponding author upon reasonable request. Source data are provided with this paper.

## Source data

Source Data
