# Supplementary material for: Programmable integrin and N-cadherin adhesive interactions modulate mechanosensing of mesenchymal stem cells by cofilin phosphorylation
Source: Nat Commun. 2022 Nov 11;13:6854. doi: 10.1038/s41467-022-34424-0 (PMC9652405; doi:10.1038/s41467-022-34424-0)
Supplement: Supplementary file 1 — Supplementary Information [file 41467_2022_34424_MOESM1_ESM.pdf]

Supplementary information for "Programmable integrin and N-cadherin adhesive interactions modulate mechanosensing of mesenchymal stem cells by cofilin phosphorylation"

Zheng Zhang[1,2], Baoyong Sha[3], Lingzhu Zhao[1,2], Huan Zhang[1,2], Jinteng Feng[1,2,4], Cheng Zhang[1,2], Lin Sun[1,2], Meiqing Luo[1,2], Bin Gao[5], Hui Guo[4], Zheng Wang[6], Feng Xu[1,2], Tian Jian Lu[7], Guy M. Genin[1,2,8,9], Min Lin[1,2#]

1 The Key Laboratory of Biomedical Information Engineering of Ministry of Education, School of Life Science and Technology, Xi'an Jiaotong University, Xi'an 710049, P.R. China
2 Bioinspired Engineering and Biomechanics Center (BEBC), Xi'an Jiaotong University, Xi'an 710049, P.R. China
3 School of Basic Medical Science, Xi'an Medical University, Xi'an 710021, P.R. China.
4 Department of Medical Oncology, First Affiliated Hospital of Xi'an Jiaotong University, Xi'an 710061, P.R. China
5 Department of Endocrinology, Second Affiliated Hospital of Air Force Military Medical University, Xi'an 710038, P.R. China
6 Department of Hepatobiliary Surgery, First Affiliated Hospital of Xi'an Jiaotong University，Xi'an 710061, P.R. China
7 State Key Laboratory of Mechanics and Control of Mechanical Structures, Nanjing University of Aeronautics and Astronautics, Nanjing 210016, P.R. China
8 Department of Mechanical Engineering & Materials Science, Washington University in St. Louis, St. Louis 63130, MO, USA
9 NSF Science and Technology Center for Engineering Mechanobiology, Washington University in St. Louis, St. Louis 63130, MO, USA

# Corresponding author: minlin@xjtu.edu.cn

This file includes:

Supplementary Texts
Supplementary Text 1 Quantification of the availability of RGD and HAVDI on the hydrogel surface.
Supplementary Text 2 Estimation of rupture force of double-stranded DNA.

Supplementary Figures
Supplementary Fig. 1 Fabrication processes for PEG hydrogels.
Supplementary Fig. 2 Synthetic scheme for the RGD-DNA1 and HAVDI-DNA2 molecules.
Supplementary Fig. 3 Cell adhesion on various coated substrates.
Supplementary Fig. 4 Kinetics of RGD conjugation and release.
Supplementary Fig. 5 Effect of the length of displacement strands on peptide release.
Supplementary Fig. 6 Measurement of peptide distributions using fluorescently tagged peptides.
Supplementary Fig. 7 State switching from "ON" to "Dual ON" beneath adherent hMSCs.
Supplementary Fig. 8 Conjugation of DNA-mediated peptides is highly sequence-specific.
Supplementary Fig. 9 Release of DNA-mediated peptides is highly sequence-specific.
Supplementary Fig. 10 The system maintains its specificity even when multiple switches were present on the same substrate.
Supplementary Fig. 11 Kinetics of HAVDI conjugation and release.
Supplementary Fig. 12 Young's moduli of the PEG hydrogels.
Supplementary Fig. 13 HAVDI do not support cell adhesion.
Supplementary Fig. 14 Effect of RGD/integrin and HAVDI/N-cadherin ligations on cell area.
Supplementary Fig. 15 Expression of integrin and N-cadherin on different substrates.
Supplementary Fig. 16 Effect of different integrin binding ligands on integrin clustering.
Supplementary Fig. 17 A partial "OFF" state attenuates integrin-driven mechanosensing.
Supplementary Fig. 18 Stiffness-dependent Lamin A expression associated with integrin and N-cadherin signaling.
Supplementary Fig. 19 RGD and HAVDI need to be tethered in order to regulate YAP nuclear localization.
Supplementary Fig. 20 Effects of DNA linkers with different rupture forces on RGD/integrin and HAVDI/N-cadherin ligations.
Supplementary Fig. 21 RGD and HAVDI signals with DNA linkers persisted for at least two weeks.
Supplementary Fig. 22 Programmable HAVDI/N-cadherin ligation regulates integrin and N-cadherin clustering.
Supplementary Fig. 23 Reversible and irreversible effects of mechanical dosing on the DNA-driven system.
Supplementary Fig. 24 Increasing RGD/integrin ligation activates mechanotransduction of hMSCs.
Supplementary Fig. 25 Decreasing HAVDI/N-cadherin ligation activates mechanotransduction of hMSCs.
Supplementary Fig. 26 Decreasing HAVDI/N-cadherin ligation regulates integrin and N-cadherin clustering.

Supplementary Fig. 27 Effect of increasing RGD and decreasing HAVDI on cell and nuclear area.
Supplementary Fig. 28 RGD/integrin and HAVDI/N-cadherin ligations regulate osteogenic differentiation of hMSCs.
Supplementary Fig. 29 Effect of RGD/integrin and HAVDI/N-cadherin ligations on nuclear area and volume.
Supplementary Fig. 30 Verification of cofilin knockdown.
Supplementary Fig. 31 Cofilin depletion promotes formation of focal adhesions in hMSCs on "OFF" and "Dual ON" substrates.
Supplementary Fig. 32 Cofilin limits the formation of actin cap and YAP nuclear localization in hMSCs on soft substrates.
Supplementary Fig. 33 Effect of RGD/integrin and HAVDI/N-cadherin ligations on profilin phosphorylation.
Supplementary Fig. 34 Effect of RGD/integrin and HAVDI/N-cadherin ligations on cofilin phosphorylation.
Supplementary Fig. 35 RGD/integrin and HAVDI/N-cadherin ligations regulate pCofilin nuclear localization.
Supplementary Fig. 36 Effect of programmable RGD/integrin and HAVDI/N-cadherin ligations on cofilin phosphorylation.

Supplementary Tables

Supplementary Table 1 Sequences of all DNA strands used.
Supplementary Table 2 Sequences of all peptides used.

Supplementary References

Supplementary Texts

Supplementary Text 1 Quantification of the availability of RGD and HAVDI on the hydrogel surface. Surface density and spacing of peptides conjugated onto PEG hydrogels were quantified using the concentrations of peptides bound to the hydrogel. From the uniform distribution of peptides observed in Supplementary Fig. 6, we evaluated the surface density (ρ) of the peptide on the hydrogels using a single-layer, cubic lattice model, so that the distance between two neighboring peptide molecules is $d = 1/\sqrt[3]{CN_{\mathrm{A}}}$, where C is the peptide concentration in the hydrogel (in mol/m^3) and N_{A} is Avogadro's number. The surface density (number of molecules per square meter) was then calculated as:

$$\rho = Cd = {N_{\mathrm{A}}}^{-1/3}(C)^{2/3} \tag{1}$$

The concentrations of RGD and HAVDI bound to the hydrogel were measured as 454 and 431 μmol·L^{-1}, respectively (Supplementary Fig. 4, 11), and the distances between the neighbor peptide molecules were 15.4 and 15.7 nm, respectively, so that the surface density of RGD was 4210 molecules·μm^{-2}, and that of HAVDI was 4070 molecules·μm^{-2}. This was in a reasonable range for cell adhesion: fibronectin polymers in a natural matrix contribute one epitope per protein copy that measures several nanometers[1]. A critical RGD nanospacing of 70 nm has been proposed, beyond which a sharp decrease of cell adhesion is observed[2, 3]. This suggests that the peptide density we used fell within a suitable regime.

Supplementary Text 2 Estimation of rupture force of double-stranded DNA. Rupture forces for double-stranded DNA (dsDNA) have been examined extensively. It was observed that the rupture force for dsDNA in an unzipping mode is independent of DNA length[4, 5]. When dsDNA is ruptured in a shear mode, the rupture force increases monotonically as dsDNA length increases and reaches a plateau at around 60~70 pN. For a dsDNA in a shear mode, the applied force is not evenly distributed on all base pairs, but only distributed on a finite number of the base pairs on both ends. This finite length is characterized as χ^{-1} and $\chi^{-1} = \sqrt{Q/2R}$, where Q is the spring constant of DNA backbone, and R is the spring constant of the bonds between base pairs. The rupture force is thus defined as[6]:

$$F = 2f_c\left[\chi^{-1}tanh\left(\chi\frac{L}{2}\right) + 1\right] \tag{2}$$

where $2f_c$ is the rupture force for one single bond, L is the base pair number of the dsDNA. Data from magnetic tweezers experiments suggest that $f_c = 3.9$ pN and $\chi^{-1} = 6.8$,[7] so that rupture forces of the 20-base pair (bp) DNA linker 1 and DNA linker 2 in a shear mode are both approximately 56 pN. Unzipping mode of dsDNA can be treated as 1 bp shear mode plus other base pairs as a thermal stabilizer, and its rupture force calculated by the above equation is 12 pN, which is in the range of 10~15 pN observed by many experiments[5, 8]. The modes and rupture forces of DNA linkers used in our study are presented in Supplementary Fig. 20a.

In our DNA linkers, the primary strands had a thiol tag at 5' or 3' terminal for tethering to a PEG substrate through thiol-maleimide bond. Then, the Peptide-DNA molecules were linked to the PEG substrates by DNA hybridization. The position of the thiol on the primary strand determines the rupture force of DNA linkers. The rupture forces for the unzipping and shear modes were therefore approximately 12 and 56 pN, respectively.

Supplementary Figures

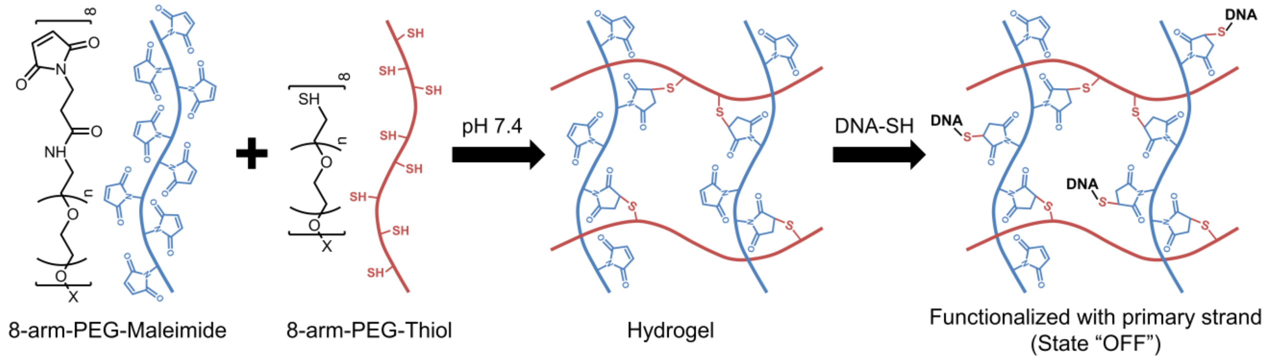

Supplementary Fig. 1 Fabrication processes for PEG hydrogels. The 8-arm PEG maleimide (PEG-MAL) and 8-arm PEG thiol (PEG-SH) were mixed to form PEG hydrogels by Michael addition reaction. The residual maleimide in the hydrogel reacted with SH-DNA to generate the primary strand-functionalized hydrogels. X = hexaglycerol core structure.

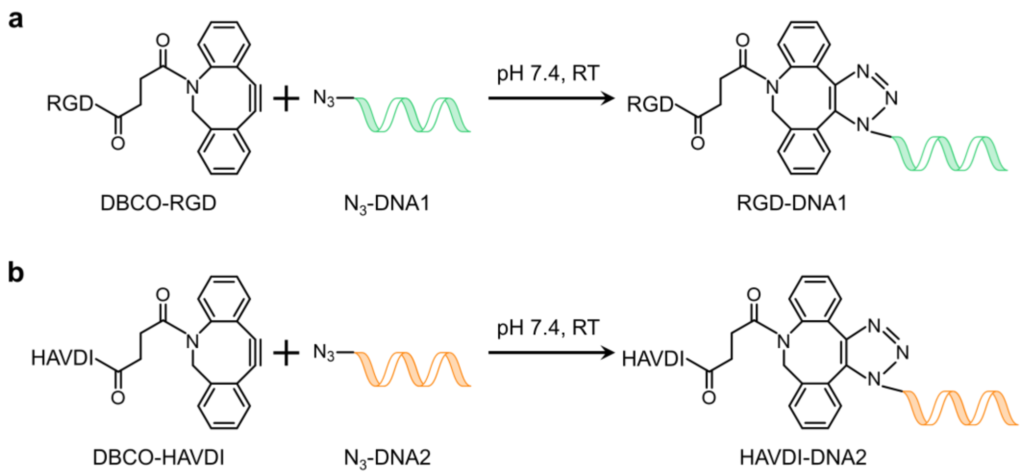

Supplementary Fig. 2 Synthetic scheme for the RGD-DNA1 and HAVDI-DNA2 molecules. a RGD was conjugated to DNA1 via copper-free click chemistry reaction between the cyclooctyne on RGD and the azide on DNA1. **b** HAVDI was conjugated to DNA2 via copper-free click chemistry reaction between the cyclooctyne on HAVDI and the azide on DNA2.

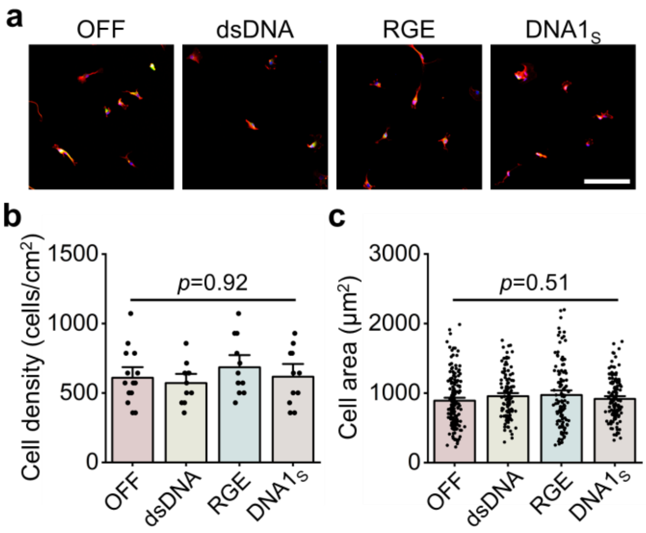

Supplementary Fig. 3 Cell adhesion on various coated substrates. a Representative immunofluorescence images in hMSCs for paxillin (green), F-actin (red) and nuclei (blue) on various coated substrates for 1 d. Corresponding quantification of **b** cell density (from left to right *n* = 15, 11, 12, 11 samples) and **c** spreading area of hMSCs (from left to right *n* = 163, 122, 113, 135 cells). hMSCs plated on substrates modified with double stranded DNA (dsDNA) without the conjugation of RGD showed poor cell numbers and spreading area analogues to "OFF" substrate. These findings suggest that the RGD is required for integrin-mediated cell adhesion. Low levels of adhesion were observed on hydrogels presenting RGE (non-bioactive peptide), confirming the specificity of the bioactive peptide RGD. "OFF" substrate was treated with RGD bound to a scrambled DNA1 sequence ($DNA1_S$) resulted in poor cell adhesion, validating that the conjugation of RGD to the hydrogels was sequence-specific. Data are presented as mean ± s.e.m., and *p* values were obtained using one-way ANOVA followed by Tukey's post hoc test (**b**, **c**). Scale bars: 100 μm (**a**). Source data are provided as a Source Data file.

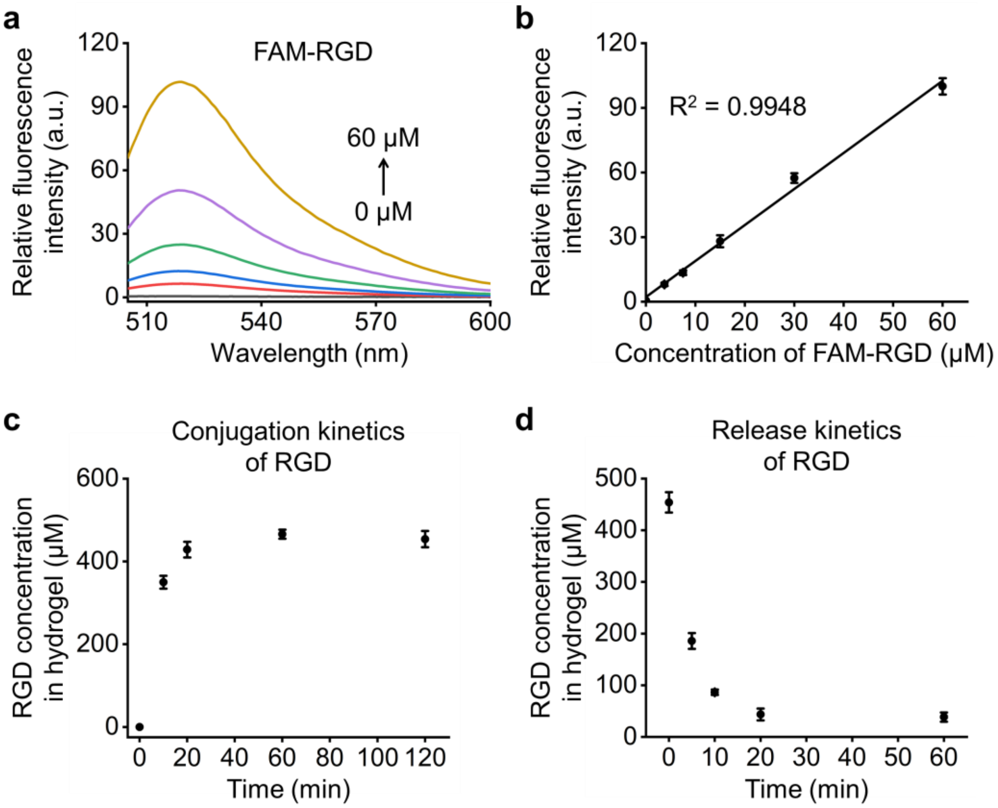

Supplementary Fig. 4 Kinetics of RGD conjugation and release. a-b Fluorescence spectrum standard curves relating fluorescence signals to the concentration of FAM-labeled RGD in solution. **a** Fluorescence spectra of the solution with 0, 3.75, 7.5, 15, 30, and 60 μM of FAM-labeled RGD. **b** Linear regression between fluorescence intensity at 522 nm and concentrations of FAM-labeled RGD (n = 3 samples per group). **c-d** According to standard curves in **b**, the conjugation and release kinetics of RGD in the PEG hydrogel could be characterized by measuring the fluorescence intensity of FAM-labeled RGD in the supernatant. **c** Conjugation kinetics of RGD as characterized after hydrogels were treated with 500 μM FAM-labeled RGD (n = 3 samples per group). **d** Release kinetics of RGD as characterized after hydrogels functionalized with FAM-labeled RGD were incubated in 500 μM solution of displacement strand 1 (n = 3 samples per group). a.u. stands for arbitrary units (**a**, **b**). Data are presented as mean ± s.e.m. (**b-d**). Source data are provided as a Source Data file.

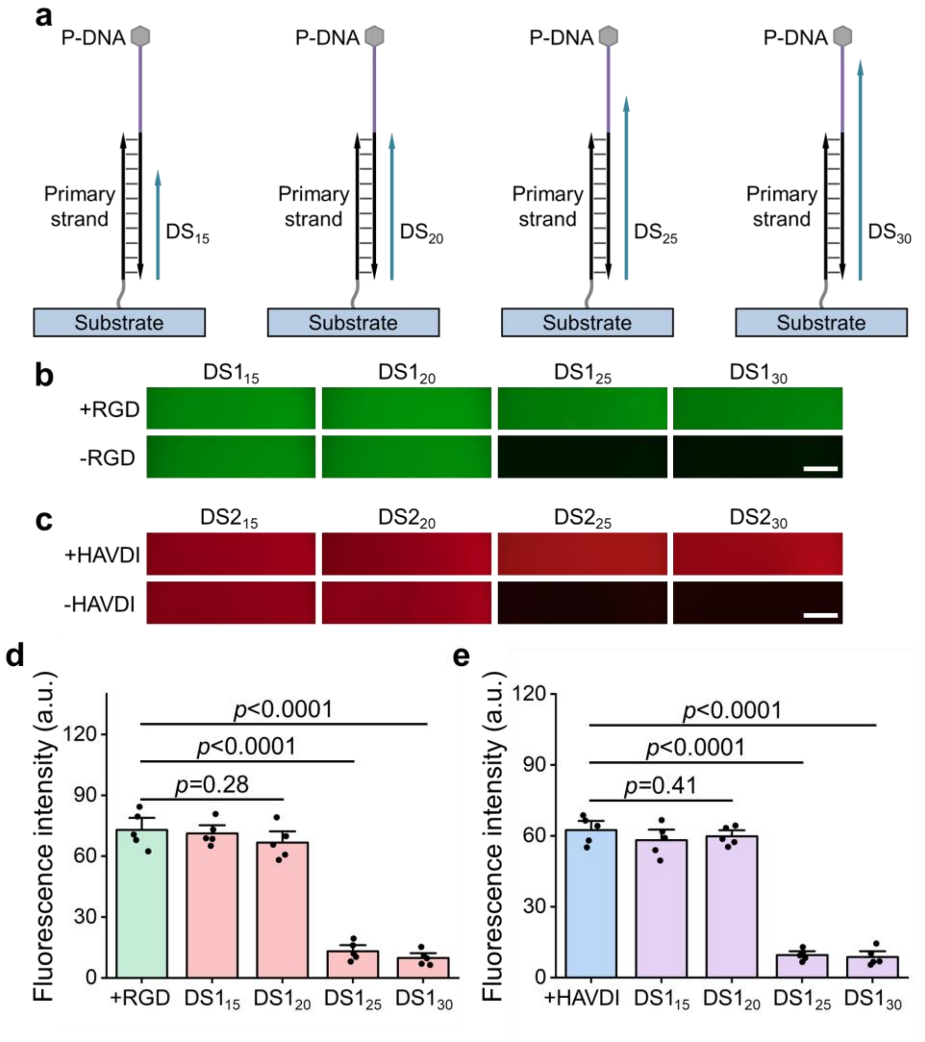

Supplementary Fig. 5 Effect of the length of displacement strands on peptide release. a Schematic diagram of complementary region of peptide-DNA and displacement strands (DS) with different lengths. The subscript numbers after DS indicates the hybridizing length. Fluorescence images of **b** FAM-labeled RGD or **c** TAMRA-labeled HAVDI conjugated to the hydrogels before and after the treatment of DS1 or DS2, respectively. Quantitative fluorescence intensity of **d** FAM-labeled RGD or **e** TAMRA-labeled HAVDI in the hydrogels (n = 5 samples per group). The sequences DS_{15} and DS_{20} did not effectively induce the two peptides release, whereas the sequences DS_{25} and DS_{30} released the two peptides from the hydrogels. Results suggested that the toehold domain is critical for peptide release. a.u. stands for arbitrary units (**d**, **e**). Data are presented as mean ± s.e.m., and p values were obtained using one-way ANOVA followed by Tukey's post hoc test (**d**, **e**). Scale bars: 100 μm (**b**, **c**). Source data are provided as a Source Data file.

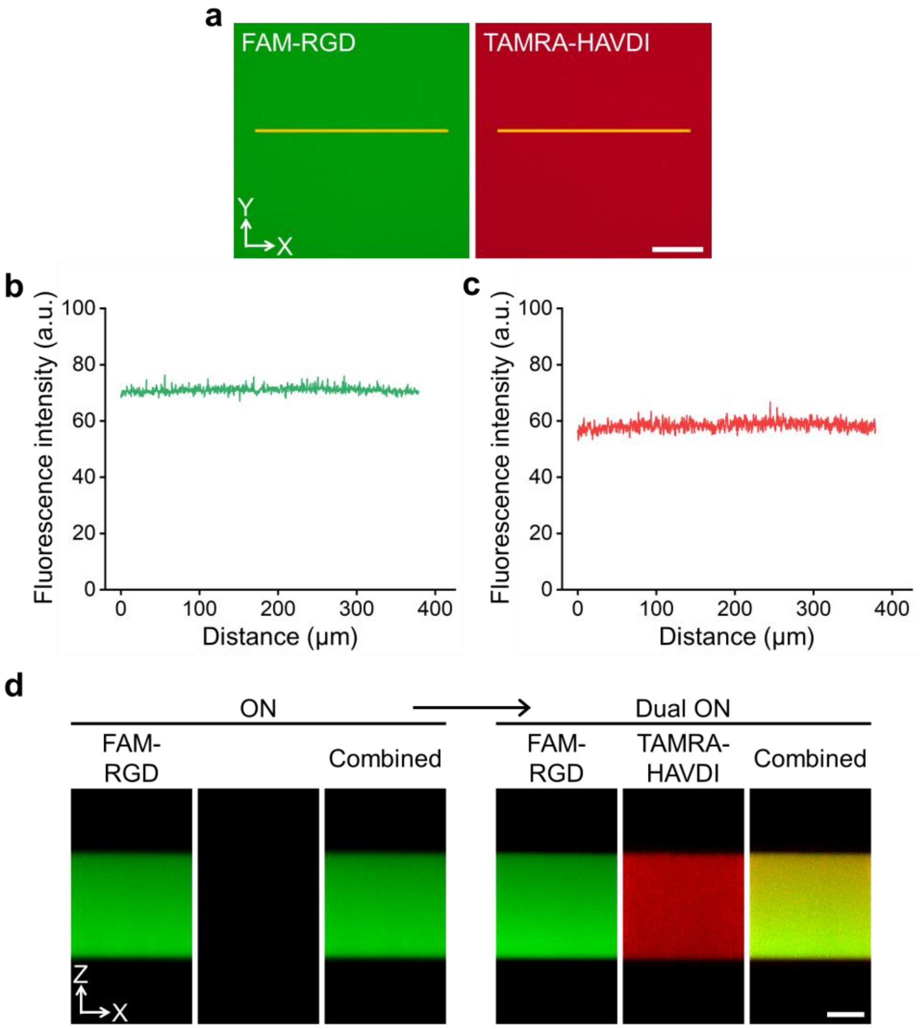

Supplementary Fig. 6 Measurement of peptide distributions using fluorescently tagged peptides. a Representative fluorescence images of hydrogels with 500 µM FAM-labeled RGD or TAMRA-labeled HAVDI (this experiment was repeated independently 3 times with similar results). Yellow lines indicate the pixel regions used to generate intensity profiles. **b-c** Fluorescence intensity profiles along the yellow lines indicate no evidence of peptide clustering in either case. **d** Representative X/Z-plane images for state switching from "ON" to "Dual ON" (this experiment was repeated independently 3 times with similar results). No phase separation between the distribution of the two peptides was observed. a.u. stands for arbitrary units (**b**, **c**). Scale bar: 100 µm (**a**), 50 µm (**d**).

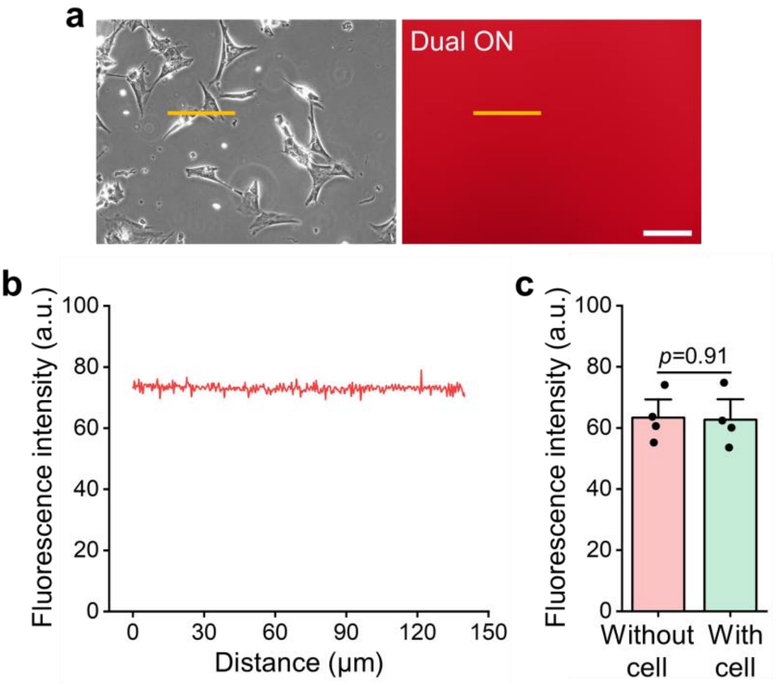

Supplementary Fig. 7 State switching from "ON" to "Dual ON" beneath adherent hMSCs. a Phase contrast and fluorescence images of an "ON" substrate surface with adherent hMSCs after adding TAMRA-labeled HAVDI for 30 min (this experiment was repeated independently 4 times with similar results). **b** Fluorescence intensity profile along the yellow line from **a**. **c** HAVDI fluorescence intensity on a hydrogel surface with and without cells (n = 4 samples per group). These results suggest that HAVDI binding to the substrate is not affected by the presence or absence of adherent cells. a.u. stands for arbitrary units (**b**, **c**). Data are presented as mean ± s.e.m., and p values were obtained using one-way ANOVA followed by Tukey's post hoc test (**c**). Scale bars: 100 μm (**a**). Source data are provided as a Source Data file.

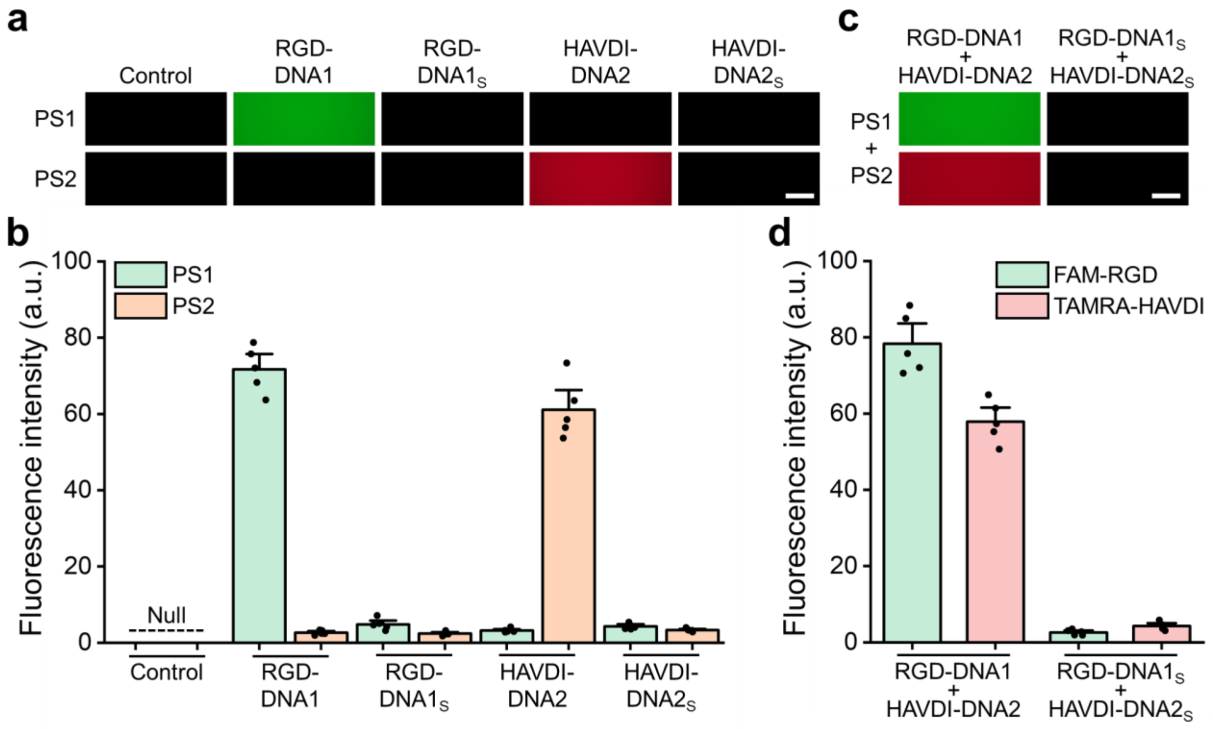

Supplementary Fig. 8 Conjugation of DNA-mediated peptides is highly sequence-specific. a Fluorescence images of hydrogels. Hydrogels functionalized with primary strand 1 (PS1) or primary strand 2 (PS2) were treated with FAM-labeled RGD-DNA1 or TAMRA-labeled HAVDI-DNA2. The subscript S indicates that $DNA1_S$ and $DNA2_S$ are scrambled sequences of DNA1 and DNA2, respectively. **b** Quantitative fluorescence intensity for the same conditions as **a** (n = 5 samples per group). **c** Hydrogels functionalized with PS1 and PS2 were treated with both of FAM-labeled RGD-DNA1 and TAMRA-labeled HAVDI-DNA2. **d** Quantitative fluorescence intensity for the same conditions as **c** (n = 5 samples per group). Hydrogels with PS1 exhibited strong fluorescence after being incubated in a solution of FAM-labeled RGD-DNA1, and hydrogels with PS2 exhibited strong fluorescence after being incubated in a solution of TAMRA-labeled HAVDI-DNA2. Results indicate that RGD and HAVDI successfully conjugated into the hydrogels. In contrast to RGD-DNA1 and HAVDI-DNA2, the fluorescence intensity did not change when the hydrogels were treated with RGD-$DNA1_S$ and HAVDI-$DNA2_S$, confirming that the conjugation of DNA-mediated peptides was sequence-specific. a.u. stands for arbitrary units (**b**, **d**). Data are presented as mean ± s.e.m. (**b**, **d**). Scale bars: 100 μm (**a**, **c**). Source data are provided as a Source Data file.

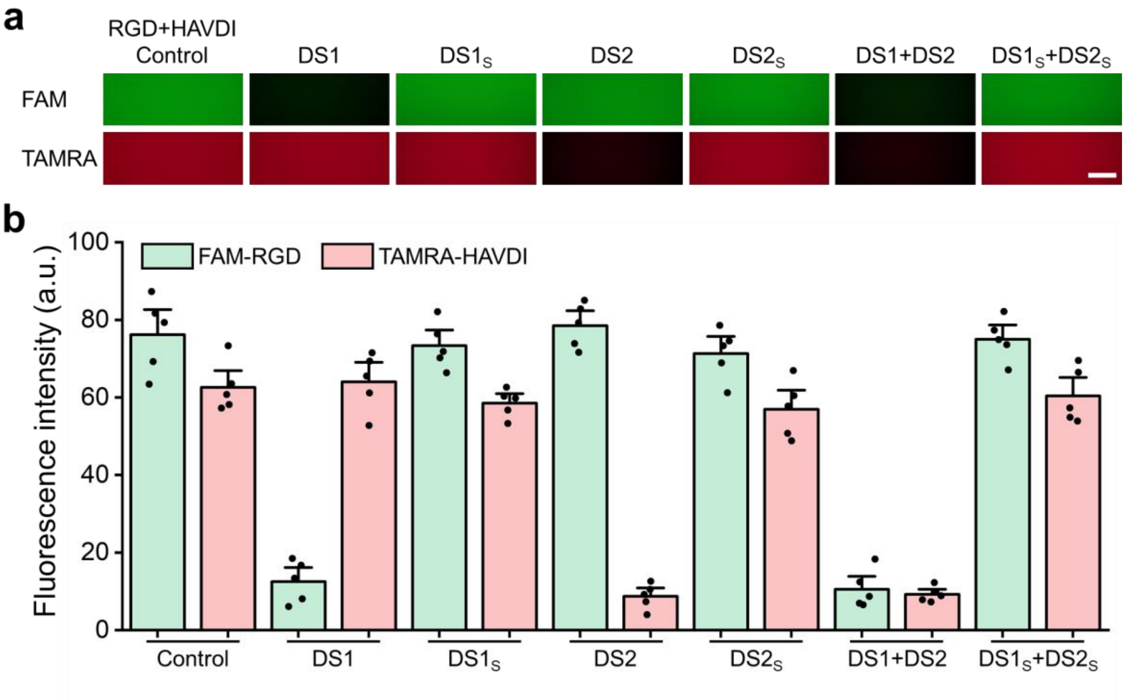

Supplementary Fig. 9 Release of DNA-mediated peptides is highly sequence-specific. a Fluorescence images of hydrogels. Hydrogels functionalized with FAM-labeled RGD and TAMRA-labeled HAVDI were treated with displacement strand 1 (DS1) or displacement strand 2 (DS2). The subscript S indicates that $DS1_S$ and $DS2_S$ are scrambled sequences of DS1 and DS2, respectively. **b** Quantitative fluorescence intensity for the same conditions as **a** (n = 5 samples per group). When the hydrogel with FAM-labeled RGD and TAMRA-labeled HAVDI was treated with DS1 and DS2, the strong fluorescence diminished. In contrast, the fluorescence intensity did not change when the hydrogels were treated with either $DS1_S$ or $DS2_S$, confirming that the release of DNA-mediated peptides was sequence-specific. a.u. stands for arbitrary units (**b**). Data are presented as mean ± s.e.m. (**b**). Scale bars: 100 μm (**a**). Source data are provided as a Source Data file.

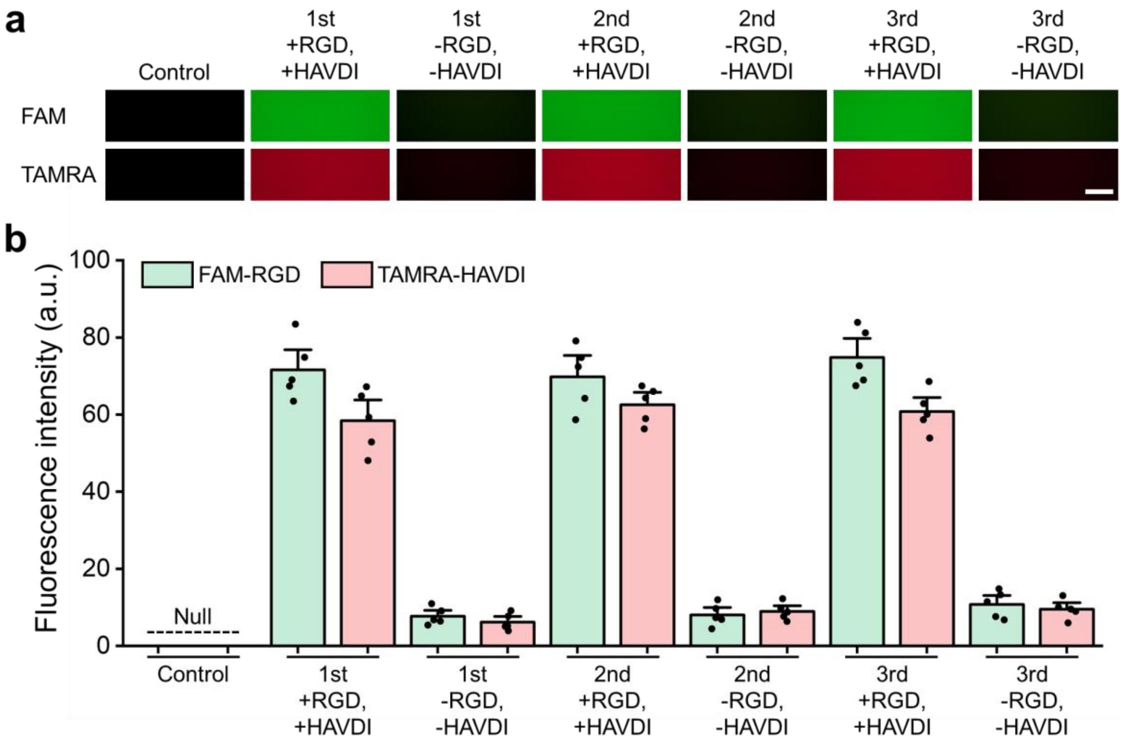

Supplementary Fig. 10 The system maintains its specificity even when multiple switches were present on the same substrate. a Representative fluorescence images of hydrogels on which FAM-labeled RGD and TAMRA-labeled HAVDI were conjugated and released over multiple cycles. **b** Quantitative fluorescence intensity of FAM-labeled RGD and TAMRA-labeled HAVDI in the hydrogels for the same conditions as **a** (n = 5 samples per group). a.u. stands for arbitrary units (**b**). Data are presented as mean ± s.e.m. (**b**). Scale bars: 100 μm (**a**). Source data are provided as a Source Data file.

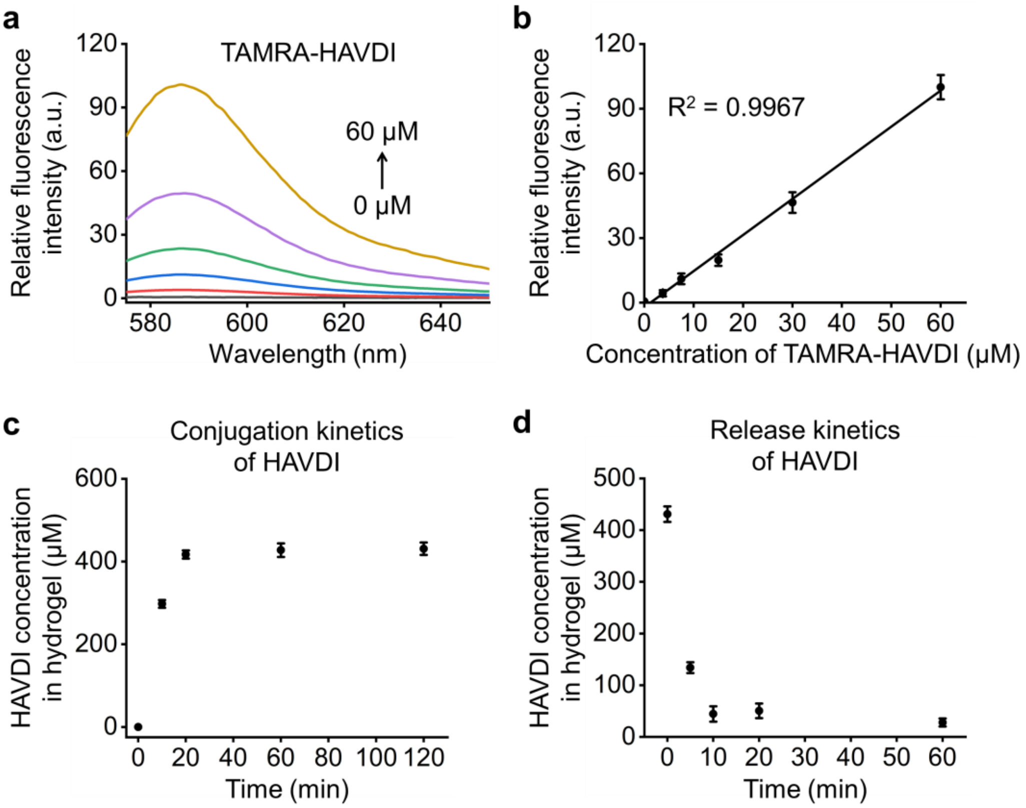

Supplementary Fig. 11 Kinetics of HAVDI conjugation and release. a-b Fluorescence spectrum standard curves relating fluorescence signals to the concentration of TAMRA-labeled HAVDI in solution. **a** Fluorescence spectra of the solution with 0, 3.75, 7.5, 15, 30, and 60 μM of TAMRA-labeled HAVDI. **b** Linear regression between fluorescence intensity at 585 nm and concentrations of TAMRA-labeled HAVDI (n = 3 samples per group). **c-d** According to standard curves in **b**, the conjugation and release kinetics of HAVDI in the PEG hydrogel could be characterized by measuring the fluorescence intensity of TAMRA-labeled HAVDI in the supernatant. **c** Conjugation kinetics of HAVDI as characterized after the hydrogels were treated with 500 μM TAMRA-labeled HAVDI (n = 3 samples per group). **d** Release kinetics of HAVDI as characterized after hydrogels functionalized with TAMRA-labeled HAVDI were incubated in 500 μM solution of displacement strand 2 (n = 3 samples per group). a.u. stands for arbitrary units (**a**, **b**). Data are presented as mean ± s.e.m. (**b-d**). Source Data are provided as a Source Data file.

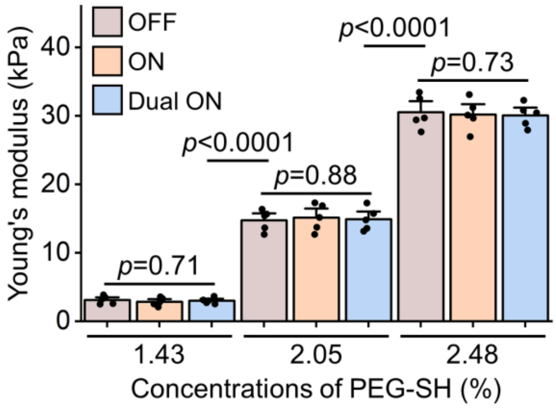

Supplementary Fig. 12 Young's moduli of the PEG hydrogels. Young's moduli of hydrogels synthesized at different concentrations of PEG-SH without or with peptide (n = 5 samples per group). Results indicate that modification of peptides do not alter the stiffness of PEG hydrogels in this system. Data are presented as mean ± s.e.m., and p values were obtained using one-way ANOVA followed by Tukey's post hoc test. Source data are provided as a Source Data file.

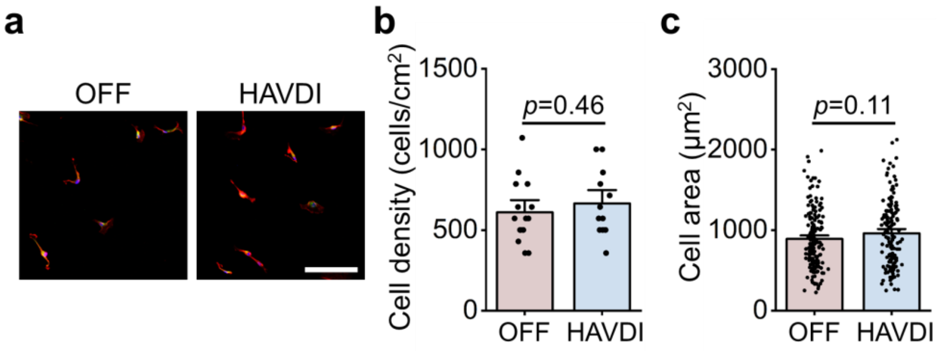

Supplementary Fig. 13 HAVDI do not support cell adhesion. **a** Representative immunofluorescence images in hMSCs for paxillin (green), F-actin (red) and nuclei (blue) on the "OFF" and HAVDI coated substrates for 1 d. **b** Corresponding quantification of cell density (from left to right n = 15, 13 samples) and **c** spreading area of hMSCs (from left to right n = 163, 147 cells). Data are presented as mean ± s.e.m., and p values were obtained using one-way ANOVA followed by Tukey's post hoc test (**b**, **c**). Scale bars: 100 μm (**a**). Source data are provided as a Source Data file.

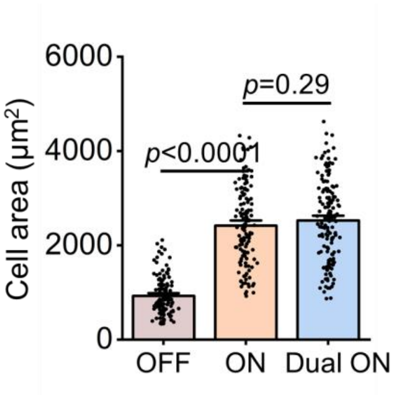

Supplementary Fig. 14 Effect of RGD/integrin and HAVDI/N-cadherin ligations on cell area. Quantification of cell area in hMSCs cultured on "OFF", "ON" or "Dual ON" substrates for 1 d (from left to right *n* = 122, 136, 153 cells). Data are presented as mean ± s.e.m., and *p* values were obtained using one-way ANOVA followed by Tukey's post hoc test. Source data are provided as a Source Data file.

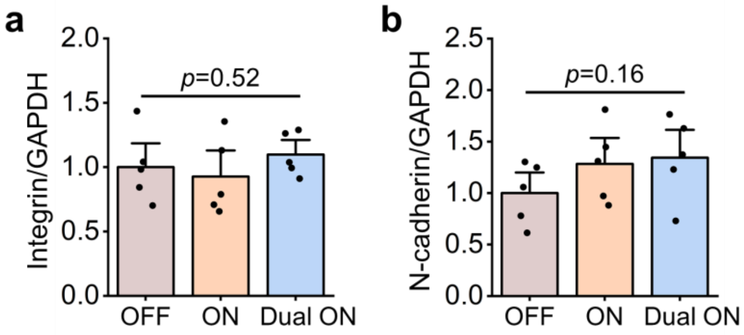

Supplementary Fig. 15 Expression of integrin and N-cadherin on different substrates. ELISA for **a** integrin and **b** N-cadherin expression in hMSCs on "OFF", "ON", "Dual ON" substrates for 2 d (n = 5 experiments per group). Data are presented as mean ± s.e.m., and p values were obtained using one-way ANOVA followed by Tukey's post hoc test (**a**, **b**). Source data are provided as a Source Data file.

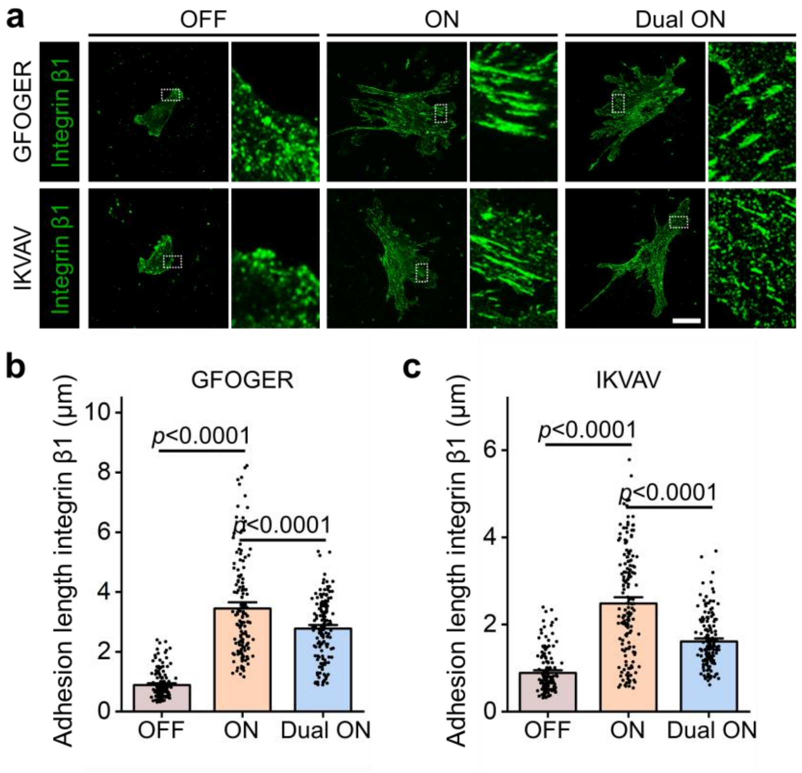

Supplementary Fig. 16 Effect of different integrin binding ligands on integrin clustering. a Top: Representative integrin β1 images in hMSCs on "OFF", "ON" and "Dual ON" substrates with GFOGER instead of RGD. Bottom: Representative integrin β1 images in hMSCs on "OFF", "ON" and "Dual ON" substrates with IKVAV instead of RGD. **b** Corresponding quantification of integrin β1 adhesion length for hMSCs in the GFOGER group (from left to right n = 146, 144, 158 adhesions in 44, 45, 49 cells respectively). **c** Corresponding quantification of integrin β1 adhesion length for hMSCs in the IKVAV group (from left to right n = 146, 167, 161 adhesions in 44, 54, 49 cells respectively). Data are presented as mean ± s.e.m., and p values were obtained using one-way ANOVA followed by Tukey's post hoc test (**b**, **c**). Scale bars: 20 μm (**a**). Source data are provided as a Source Data file.

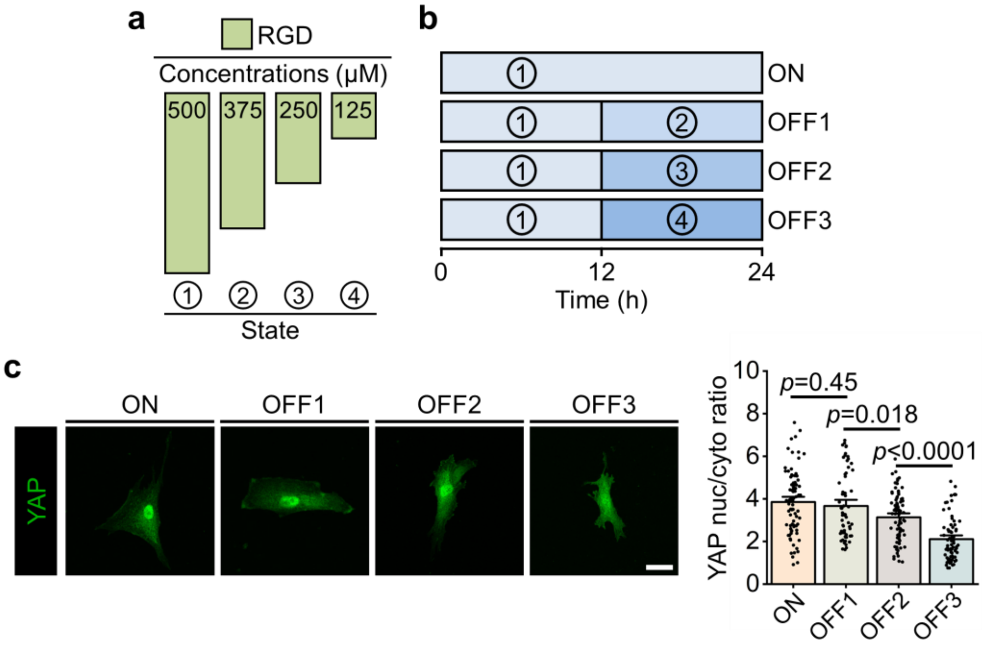

Supplementary Fig. 17 A partial "OFF" state attenuates integrin-driven mechanosensing. a Schematic of the protocol for achieving a partial "OFF" state through addition of varying concentrations of displacement strand 1. Concentration of RGD in the substrate was initially 500 μM, and then the different concentrations of displacement strand 1 (125, 250, 375 μM) were added. **b** Concentration of RGD was varied at 12 h for the same conditions as **a**, until a total culture time of 24 h. **c** Left: Representative YAP images in hMSCs for the same conditions as **b**. Right: Corresponding quantification of YAP nuc/cyto ratios (from left to right n = 85, 64, 81, 76 cells). Data are presented as mean ± s.e.m., and p values were obtained using one-way ANOVA followed by Tukey's post hoc test (**c**). Scale bars: 30 μm (**c**). Source data are provided as a Source Data file.

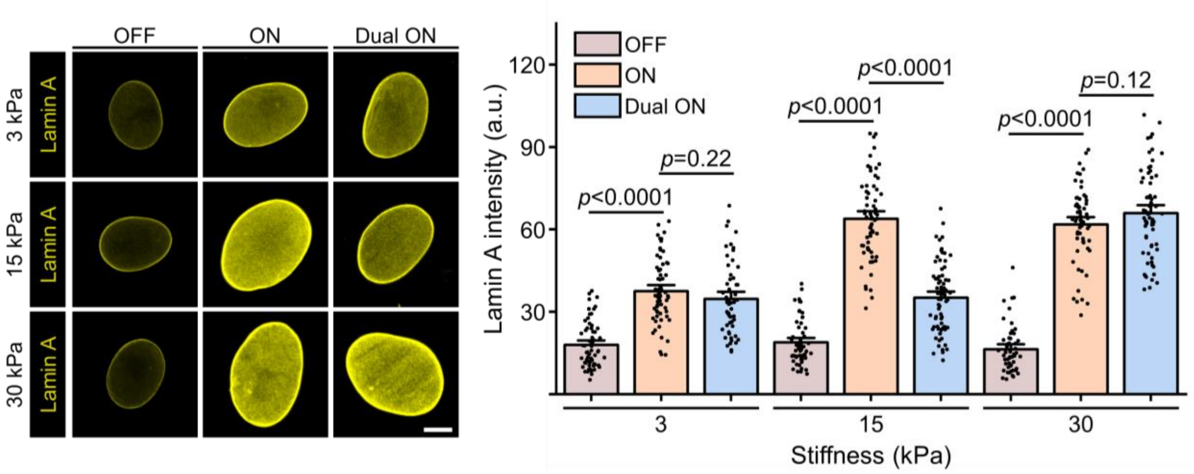

Supplementary Fig. 18 Stiffness-dependent Lamin A expression associated with integrin and N-cadherin signaling. Left: Representative Lamin A images in hMSCs on 3, 15 and 30 kPa of "OFF", "ON" or "Dual ON" substrates for 1 d. Right: Corresponding quantification of Lamin A intensity (from left to right n = 61, 64, 56, 57, 65, 72, 52, 61, 68 cells). Data are presented as mean ± s.e.m., and p values were obtained using one-way ANOVA followed by Tukey's post hoc test. Scale bars: 5 µm. Source data are provided as a Source Data file.

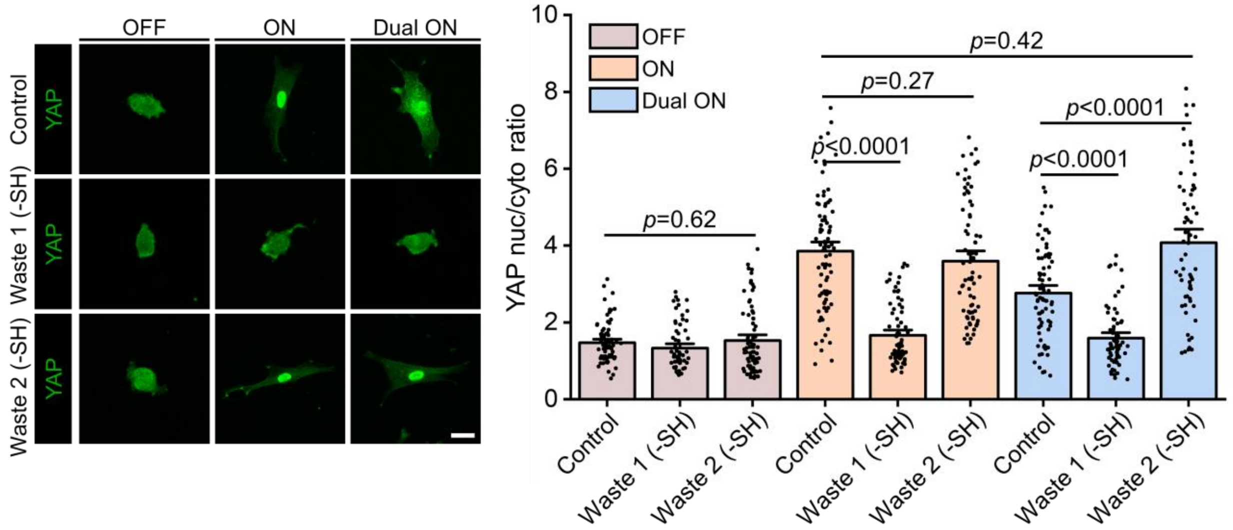

Supplementary Fig. 19 RGD and HAVDI need to be tethered in order to regulate YAP nuclear localization. Left: Representative YAP images in hMSCs on “OFF”, “ON” or “Dual ON” substrates for 1 d, and followed by treatment with 500 μM of waste 1 or waste 2 (without thiol group, referred to as “-SH”). Waste 1 and 2 block integrin and N-cadherin binding, respectively. No waste treatment as control groups. Right: Corresponding quantification of YAP nuc/cyto ratios (from left to right n = 74, 65, 77, 85, 76, 75, 81, 68, 62 cells). hMSCs on “ON” substrate showed a significant decrease in YAP nuc/cyto ratios when treated with integrin blocking waste 1 (-SH), as did hMSCs on “OFF” substrate. hMSCs on “Dual ON” substrate showed a significant increase in YAP nuc/cyto ratios when treated with N-cadherin blocking waste 2 (-SH), as did hMSCs on “ON” substrate. Data are presented as mean ± s.e.m., and p values were obtained using one-way ANOVA followed by Tukey’s post hoc test. Scale bars: 30 μm. Source data are provided as a Source Data file.

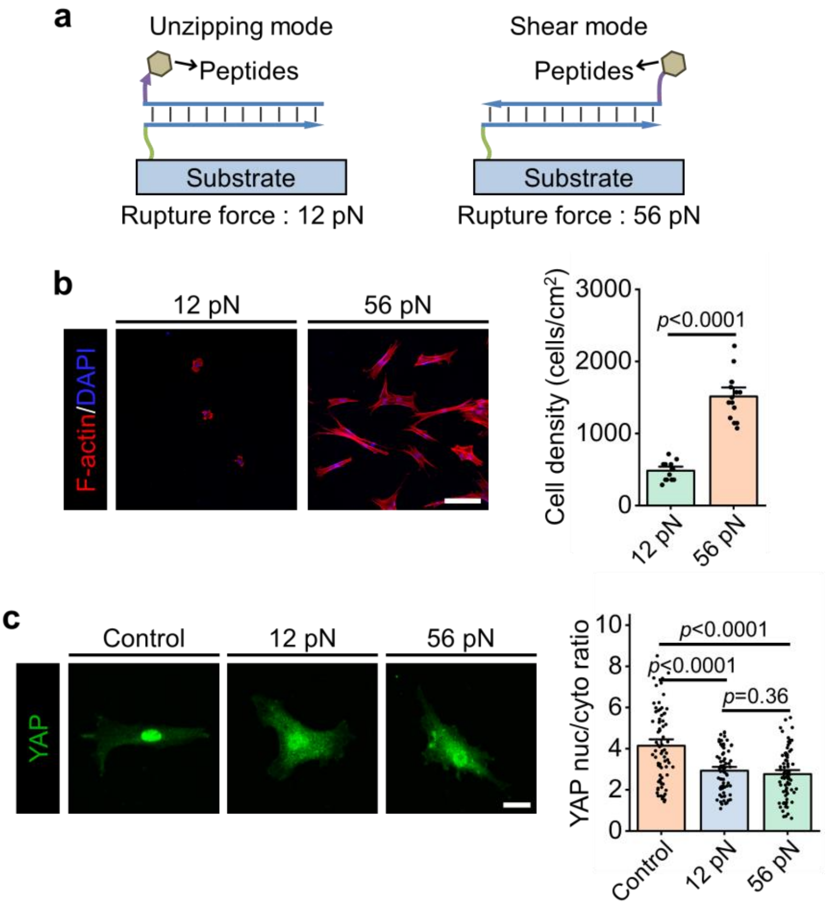

Supplementary Fig. 20 Effects of DNA linkers with different rupture forces on RGD/integrin and HAVDI/N-cadherin ligations. a Rupture force of DNA linkers depends on the force anchoring positions. The estimated rupture force of DNA linkers is 12 pN for the unzipping mode (left) and 56 pN for the shear mode (right). **b** Left: Representative images (red: F-actin, blue: DAPI) in hMSCs on "ON" substrates for 1 d on which DNA linker 1 (RGD signals) had rupture forces of 12 or 56 pN. Right: Corresponding quantification of cell density (from left to right *n* = 13, 15 samples). **c** Left: Representative YAP images in hMSCs on "Dual ON" substrates for 1 d on which DNA linker 2 (HAVDI signals) had rupture forces of 12 or 56 pN. Cells were cultured on "ON" substrates with HAVDI-DNA2$_S$ as control groups. The subscript S indicates that DNA2$_S$ is scrambled sequences of DNA2. Right: Corresponding quantification of YAP nuc/cyto ratios (from left to right *n* = 74, 71, 81 cells). Data are presented as mean ± s.e.m., and *p* values were obtained using one-way ANOVA followed by Tukey's post hoc test (**b**, **c**). Scale bars: 100 μm (**b**), 30 μm (**c**). Source data are provided as a Source Data file.

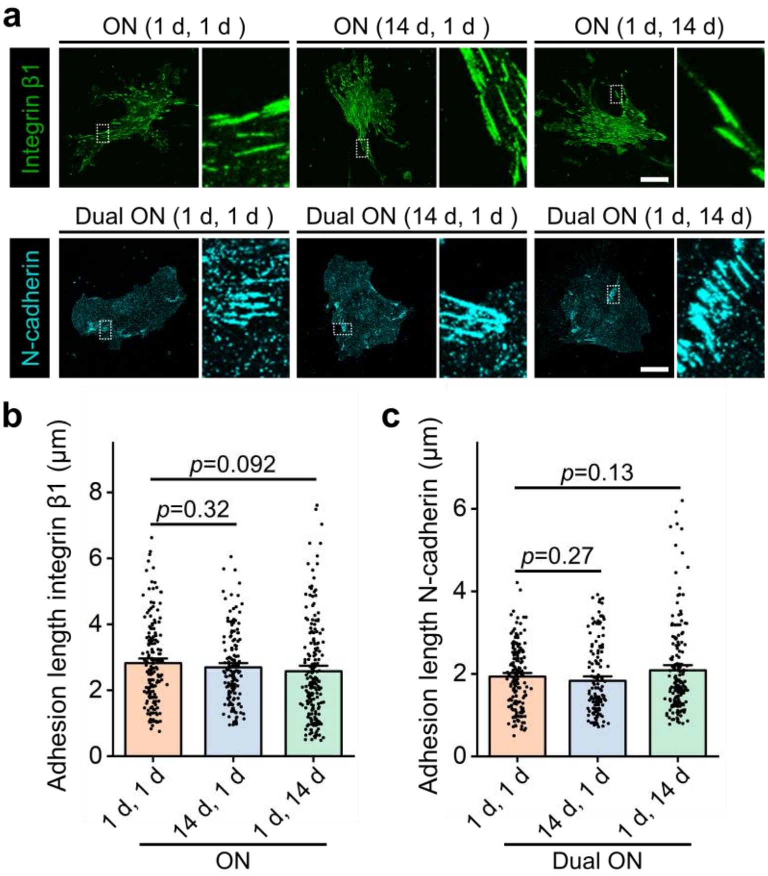

Supplementary Fig. 21 RGD and HAVDI signals with DNA linkers persisted for at least two weeks. a Top: Representative integrin β1 images in hMSCs on "ON" substrates for a prescribed time. Bottom: Representative N-cadherin images in hMSCs on "Dual ON" substrates for a prescribed time. Number of days (X) that the substrates were sat in growth medium without cells and number of days (Y) that hMSCs were cultured on the substrates was referred to as "X d, Y d". **b** Corresponding quantification of integrin β1 adhesion length for the same conditions as **a** (from left to right n = 172, 157, 186 adhesions in 53, 51, 58 cells respectively). **c** Corresponding quantification of N-cadherin adhesion length for the same conditions as **a** (from left to right n = 166, 145, 174 adhesions in 51, 47, 55 cells respectively). Data are presented as mean ± s.e.m., and p values were obtained using one-way ANOVA followed by Tukey's post hoc test (**b**, **c**). Scale bars: 20 μm (**a**). Source data are provided as a Source Data file.

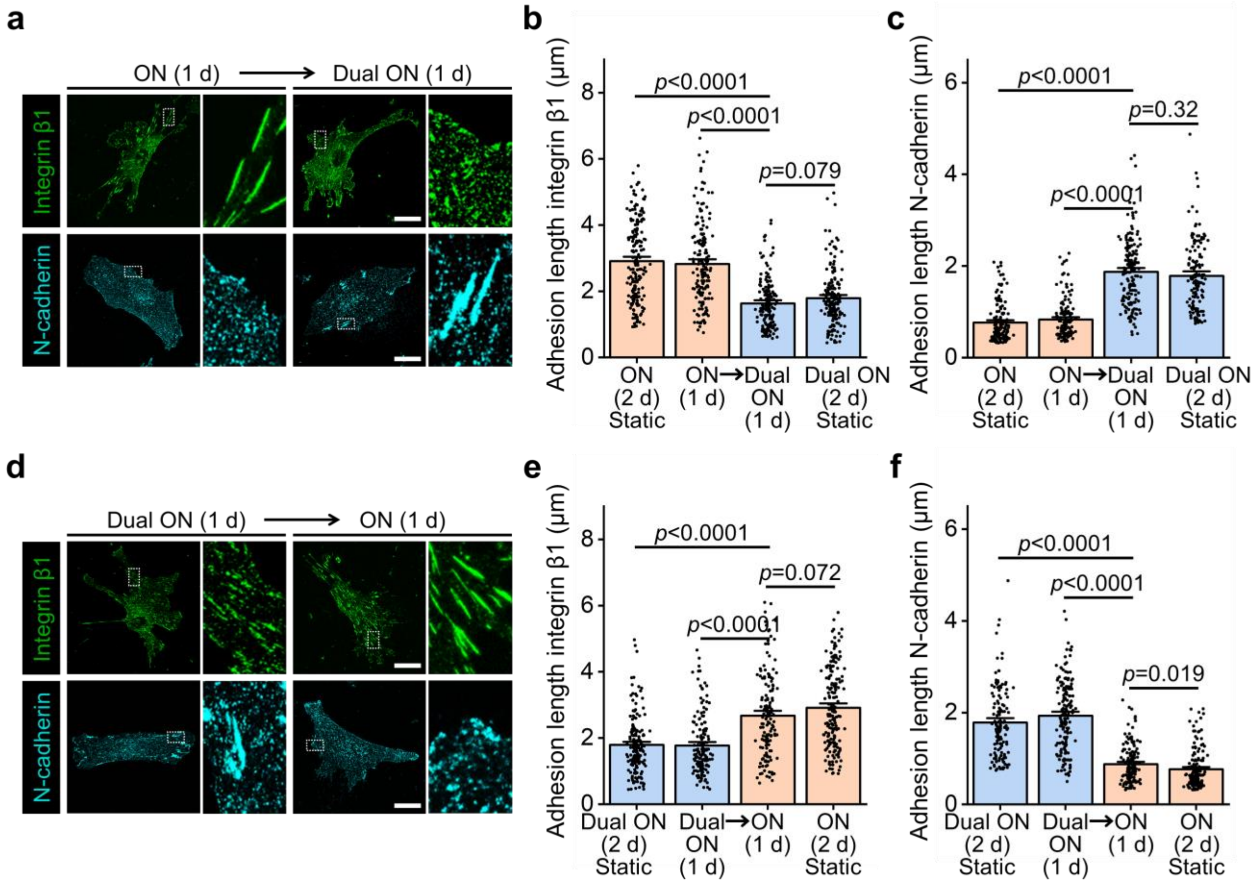

Supplementary Fig. 22 Programmable HAVDI/N-cadherin ligation regulates integrin and N-cadherin clustering. a Representative integrin β1 and N-cadherin images in hMSCs for state switching from "ON" for initial 1 d to "Dual ON" for subsequent 1 d. **b** Corresponding quantification of integrin β1 adhesion length for the same conditions as **a** (from left to right n = 184, 172, 149, 174 adhesions in 59, 53, 46, 56 cells respectively). Cells were cultured on continuously "ON" and "Dual ON" substrates for 2 d as control groups. **c** Corresponding quantification of N-cadherin adhesion length for the same conditions as **a** (from left to right n = 156, 144, 164, 139 adhesions in 50, 45, 53, 42 cells respectively). Cells were cultured on continuously "ON" and "Dual ON" substrates for 2 d as control groups. **d** Representative integrin β1 and N-cadherin images in hMSCs for state switching from "Dual ON" for initial 1 d to "ON" for subsequent 1 d. **e** Corresponding quantification of integrin β1 adhesion length for the same conditions as **d** (from left to right n = 174, 159, 152, 184 adhesions in 56, 48, 51, 59 cells respectively). Cells were cultured on continuously "Dual ON" and "ON" substrates for 2 d as control groups. **f** Corresponding quantification of N-cadherin adhesion length for the same conditions as **d** (from left to right n = 139, 166, 142, 156 adhesions in 42, 51, 46, 50 cells respectively). Cells were cultured on continuously

“Dual ON” and “ON” substrates for 2 d as control groups. Data are presented as mean ± s.e.m., and *p* values were obtained using one-way ANOVA followed by Tukey’s post hoc test (**b**, **c**, **e**, **f**). Scale bars: 20 μm (**a**, **d**). Source data are provided as a Source Data file.

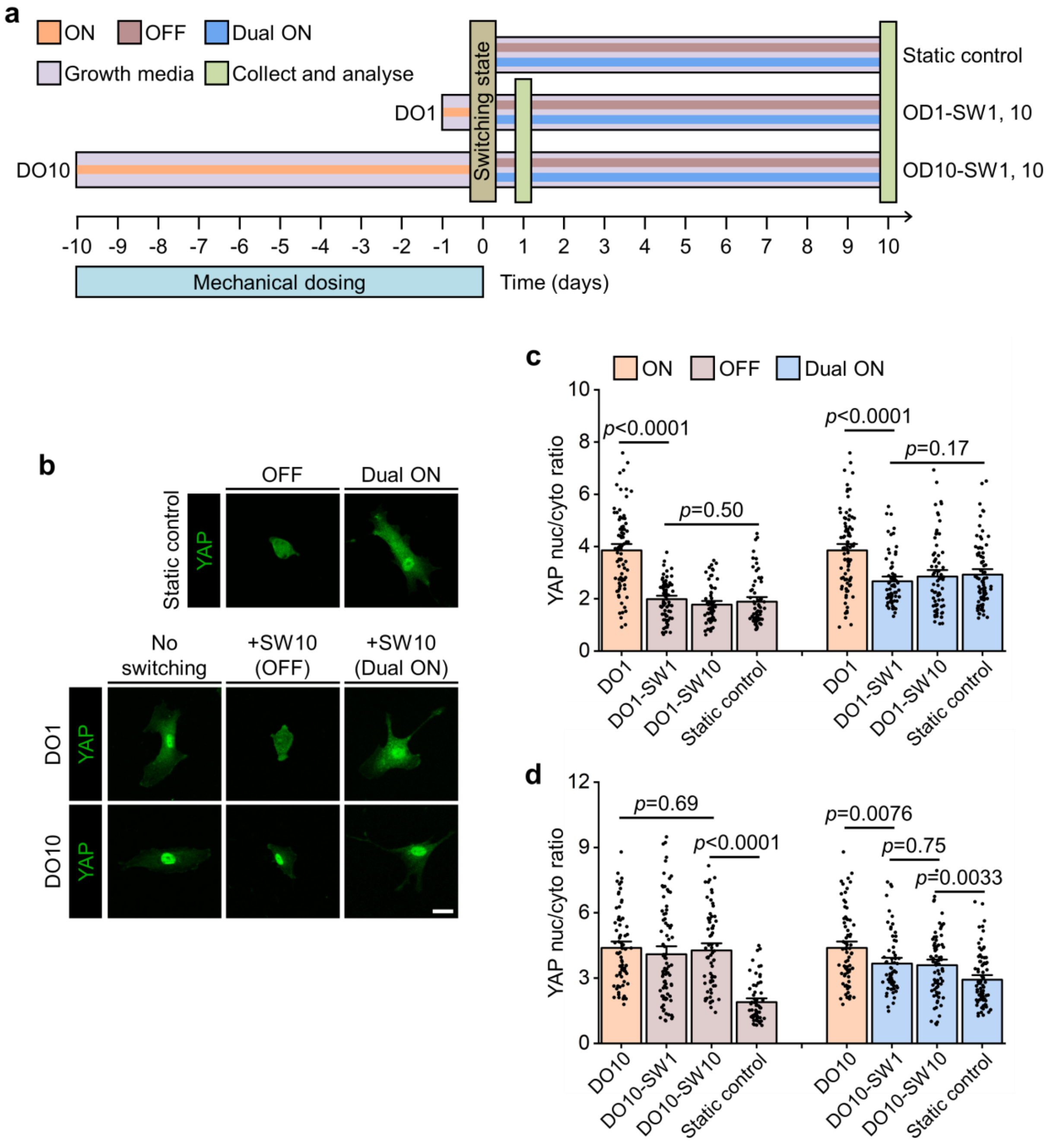

Supplementary Fig. 23 Reversible and irreversible effects of mechanical dosing on the DNA-driven system. a hMSCs were cultured on "ON" substrate (orange) in growth media for 1 or 10 days (referred to as "DO1" and "DO10") before *in situ* switching (day 0) the substrate state to "OFF" (brown) or "Dual ON" (blue). hMSCs were cultured subsequently on the substrates of switching state for 1 or 10 days before collection and analysis (green). These conditions were referred to as "DO1-SW1, 10" or "DO10-SW1, 10". Cells were cultured on continuously "OFF" or "Dual ON" substrates for 10 d as control groups (referred to as "static control"). **b** Representative YAP images in hMSC for the same conditions as **a**. **c-d** Corresponding quantification of YAP

nuc/cyto ratios in hMSCs for the same conditions as **a**. “OFF” static control and “Dual ON” static control served as baselines for YAP nuc/cyto ratios after 10 d of culture on “OFF” and “Dual ON” substrates, respectively. **c** After 1 d of culture on “ON” substrate (DO1), YAP localized to the nucleus. Following state switching to either “OFF” or “Dual ON” and culture for 1 d or 10 days (DO1-SW1, 10), the YAP nuc/cyto ratios decreased separately to the baseline levels of “OFF” or “Dual ON” static control (from left to right n = 85, 73, 62, 65, 85, 67, 71, 79 cells), showing fully reversible YAP nuclear accumulation. **d** Following 10 d of culture on “ON” substrate (DO10), YAP nuc/cyto ratios remained unchanged after state switching to “OFF” for up to 10 d (“ON-OFF”: DO10-SW1, 10), but YAP nuc/cyto ratios decreased towards baseline levels and maintained the levels after state switching to “Dual ON” for up to 10 d (“ON-Dual ON”: DO10-SW1, 10) (from left to right n = 74, 84, 68, 65, 74, 64, 72, 79 cells). Data are presented as mean ± s.e.m., and p values were obtained using one-way ANOVA followed by Tukey’s post hoc test (**c**, **d**). Scale bars: 30 μm (**b**). Source data are provided as a Source Data file.

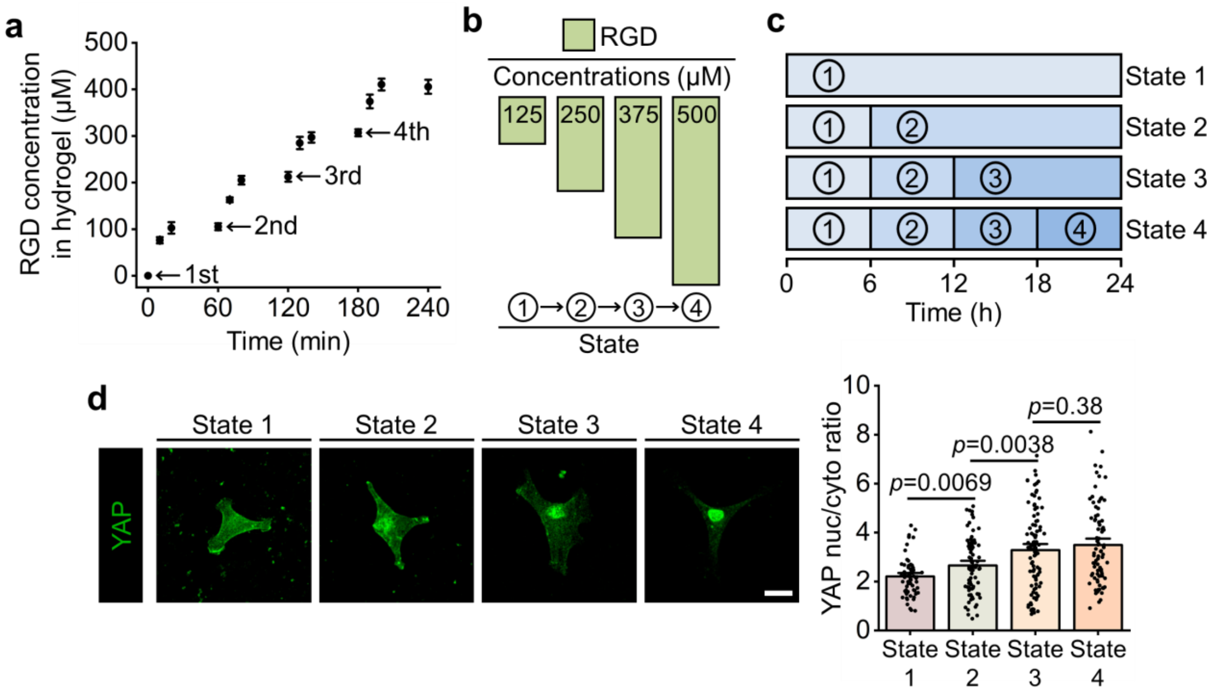

Supplementary Fig. 24 Increasing RGD/integrin ligation activates mechanotransduction of hMSCs. a According to standard curves in **Supplementary Fig. 4b**, the conjugation and release kinetics of RGD in the PEG hydrogel could be characterized by measuring the fluorescence intensity of FAM-labeled RGD in the supernatant. Concentrations of RGD gradually conjugated to the substrates following four different additions of 125 μM RGD-DNA1 (at times 0, 60, 120, and 180 min), revealing similar kinetics over all four additions (*n* = 3 samples per group). **b** Schematic of the protocol for achieving increasing RGD presentation on the substrates. Concentration of RGD in the substrate was initially 125 μM, and then the same concentration of RGD-DNA1 (125 μM) was added every time. **c** Concentration of RGD was varied every 6 h for the same conditions as **b**, until a total culture time of 24 h. **d** Left: Representative YAP images in hMSCs for the same conditions as **c**. Right: Corresponding quantification of YAP nuc/cyto ratios (from left to right *n* = 68, 82, 96, 85 cells). Data are presented as mean ± s.e.m., and *p* values were obtained using one-way ANOVA followed by Tukey's post hoc test (**a**, **d**). Scale bars: 30 μm (**d**). Source data are provided as a Source Data file.

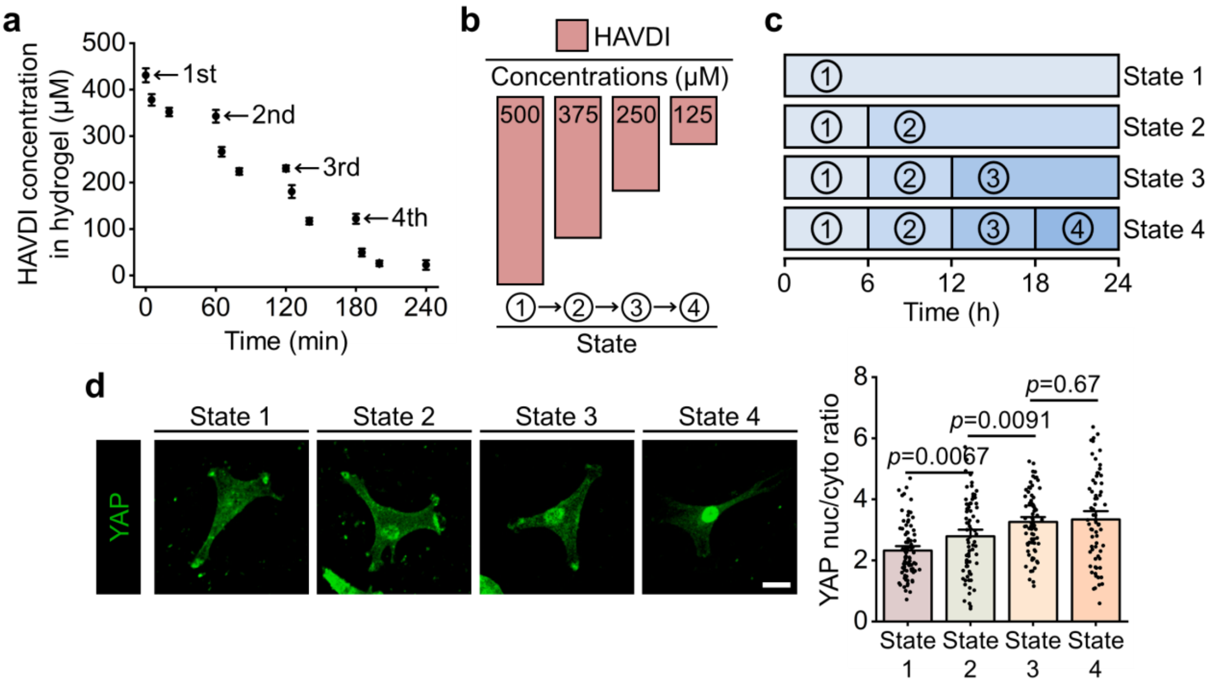

Supplementary Fig. 25 Decreasing HAVDI/N-cadherin ligation activates mechanotransduction of hMSCs. a According to standard curves in **Supplementary Fig. 11b**, the conjugation and release kinetics of HAVDI in the PEG hydrogel could be characterized by measuring the fluorescence intensity of TAMRA-labeled HAVDI in the supernatant. Concentrations of HAVDI gradually released from the substrates following four different additions of 125 μM displacement strand 2 (at times 0, 60, 120, and 180 min), revealing similar kinetics over all four additions (n = 3 samples per group). **b** Schematic of the protocol for achieving decreasing HAVDI presentation (in the context of constant RGD ligand, 500 μM) on the substrates. Concentration of HAVDI in the substrate was initially 500 μM, and then the same concentration of displacement strand 2 (125 μM) was added every time to release the HAVDI from the substrate. **c** Concentration of HAVDI was varied every 6 h for the same conditions as **b**, until a total culture time of 24 h. **d** Left: Representative YAP images in hMSCs for the same conditions as **c**. Right: Corresponding quantification of YAP nuc/cyto ratios (from left to right n = 86, 73, 82, 67 cells). Data are presented as mean ± s.e.m., and p values were obtained using one-way ANOVA followed by Tukey's post hoc test (**a**, **d**). Scale bars: 30 μm (**d**). Source data are provided as a Source Data file.

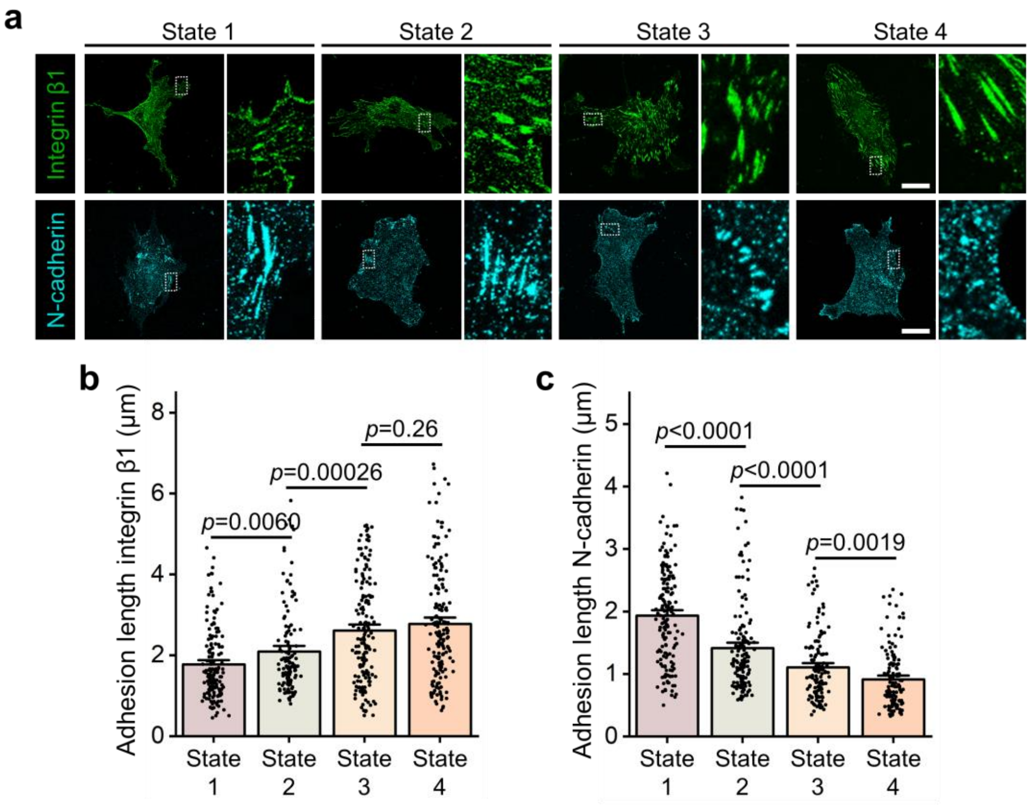

Supplementary Fig. 26 Decreasing HAVDI/N-cadherin ligation regulates integrin and N-cadherin clustering. a Representative integrin β1 and N-cadherin images in hMSCs for the same conditions as **Supplementary Fig. 25d**. **b** Corresponding quantification of integrin β1 adhesion length for the same conditions as **a** (from left to right n = 159, 131, 162, 170 adhesions in 48, 42, 51, 54 cells respectively). **c** Corresponding quantification of N-cadherin adhesion length for the same conditions as **a** (from left to right n = 166, 152, 133, 136 adhesions in 51, 47, 42, 43 cells respectively). Data are presented as mean ± s.e.m., and p values were obtained using one-way ANOVA followed by Tukey's post hoc test (**b**, **c**). Scale bars: 20 μm (**a**). Source data are provided as a Source Data file.

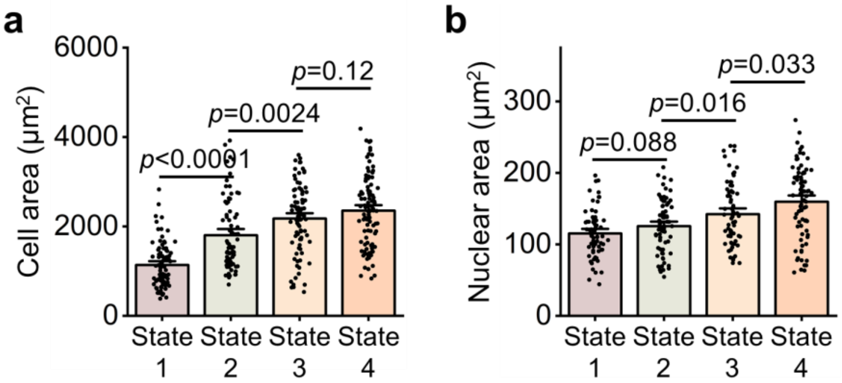

Supplementary Fig. 27 Effect of increasing RGD and decreasing HAVDI on cell and nuclear area. a Quantification of spreading area in hMSCs for the same conditions as **Fig. 3h** (from left to right n = 82, 76, 89, 97 cells). **b** Quantification of projected nuclear area in hMSCs for the same conditions as **Fig. 3h** (from left to right n = 65, 75, 67, 78 cells). Data are presented as mean ± s.e.m., and p values were obtained using one-way ANOVA followed by Tukey's post hoc test (**a**, **b**). Source data are provided as a Source Data file.

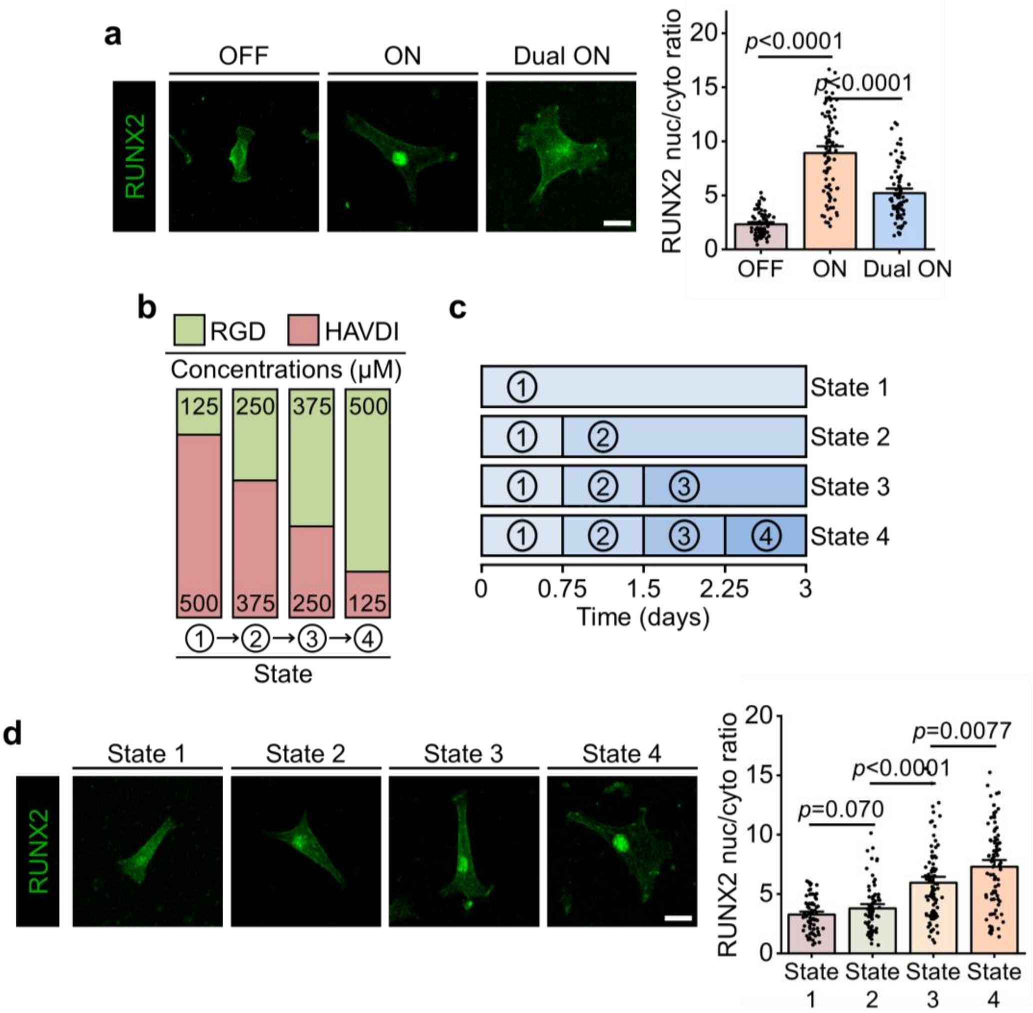

Supplementary Fig. 28 RGD/integrin and HAVDI/N-cadherin ligations regulate osteogenic differentiation of hMSCs. a Left: Representative RUNX2 images in hMSCs on "OFF", "ON" or "Dual ON" substrates for 3 d in osteoinductive media. Right: Corresponding quantification of RUNX2 nuc/cyto ratios (from left to right n = 67, 85, 77 cells). **b** Schematic of the protocol for simultaneously increasing RGD presentation and decreasing HAVDI presentation on the substrates to mimic the evolution of the mechanical microenvironment during mesenchymal development. **c** Concentrations of RGD and HAVDI were simultaneously varied every 0.75 d for the same conditions as **b**, until a total culture time of 3 d. **d** Left: Representative RUNX2 images in hMSCs for the same conditions as **c**. Right: Corresponding quantification of RUNX2 nuc/cyto ratios (from left to right n = 76, 72, 93, 84 cells). Data are presented as mean ± s.e.m., and p values were obtained using one-way ANOVA followed by Tukey's post hoc test (**a**, **d**). Scale bars: 30 μm (**a**, **d**). Source data are provided as a Source Data file.

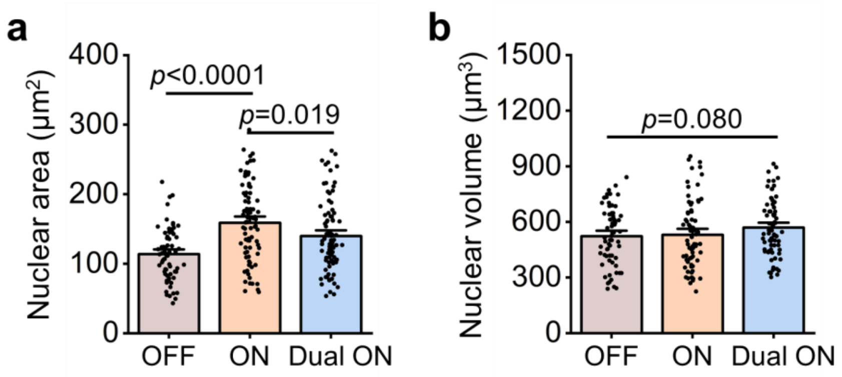

Supplementary Fig. 29 Effect of RGD/integrin and HAVDI/N-cadherin ligations on nuclear area and volume. a Quantification of nuclear area in hMSCs for the same conditions as Fig. 4c (from left to right n = 69, 84, 87 cells). **b** Quantification of nuclear volume in hMSCs for the same conditions as Fig. 4c (from left to right n = 65, 72, 74 cells). Data are presented as mean ± s.e.m., and p values were obtained using one-way ANOVA followed by Tukey's post hoc test (**a**, **b**). Source data are provided as a Source Data file.

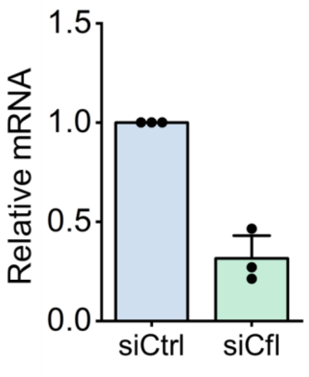

Supplementary Fig. 30 Verification of cofilin knockdown. Relative mRNA levels of cofilin determined by qRT-PCR when hMSCs were transfected with siRNAs targeting cofilin after 2 d of culture (n = 3 experiments per group). Data are presented as mean ± s.e.m.. Source data are provided as a Source Data file.

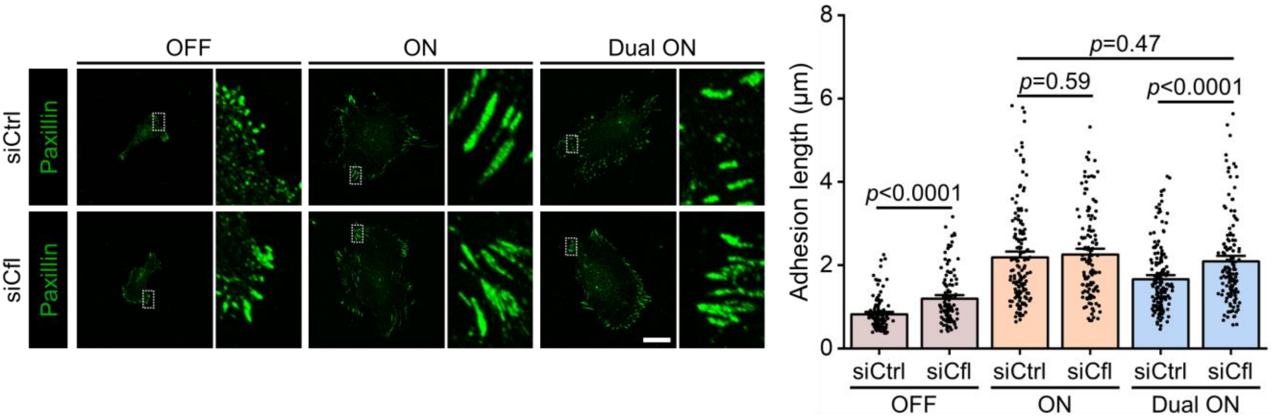

Supplementary Fig. 31 Cofilin depletion promotes formation of focal adhesions in hMSCs on “OFF” and “Dual ON” substrates. Left: Representative paxillin images in hMSCs on “OFF”, “ON” and “Dual ON” substrates for 1 d, with or without cofilin depletion. Right: Corresponding quantification of paxillin adhesion length (from left to right n = 107, 119, 147, 131, 154, 142 adhesions in 33, 38, 45, 40, 48, 45 cells respectively). Data are presented as mean ± s.e.m., and p values were obtained using one-way ANOVA followed by Tukey’s post hoc test. Scale bars: 20 μm. Source data are provided as a Source Data file.

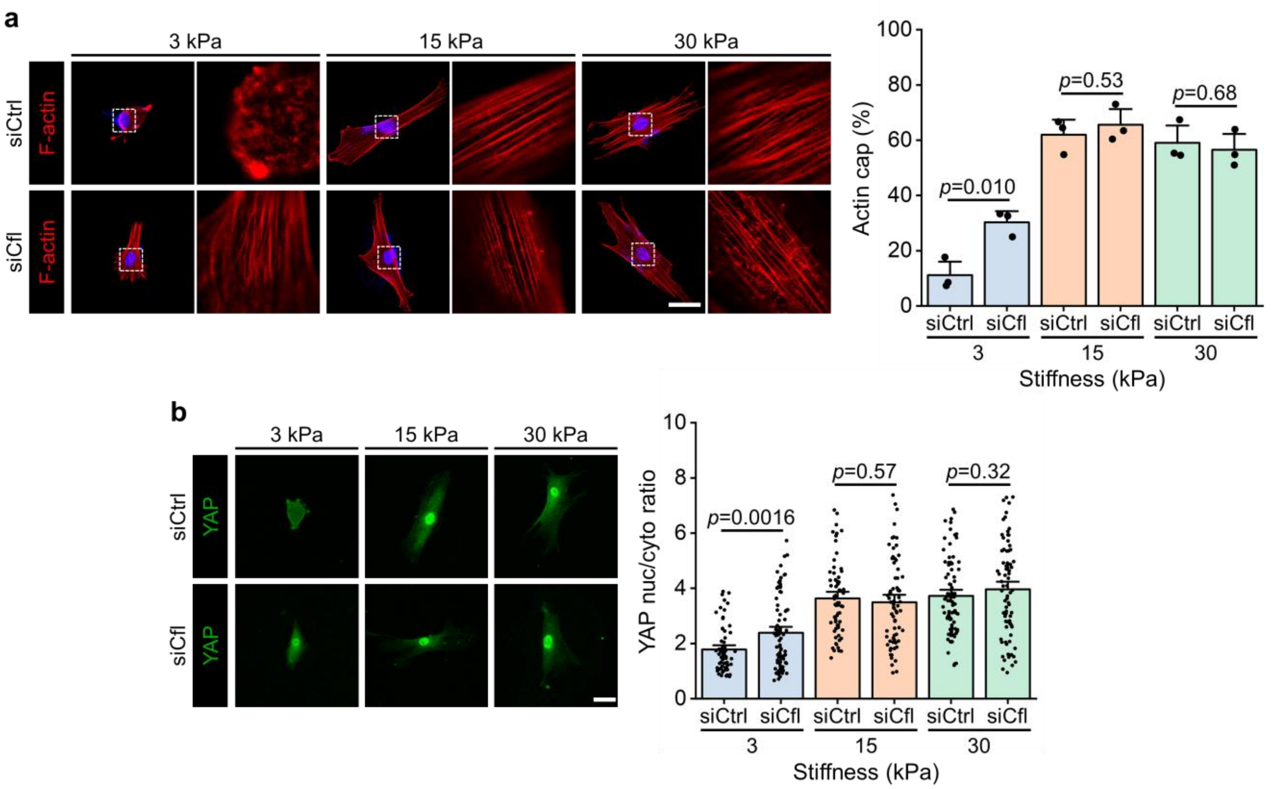

Supplementary Fig. 32 Cofilin limits the formation of actin cap and YAP nuclear localization in hMSCs on soft substrates. a Left: Representative F-actin images in control and cofilin-depleted hMSCs on "ON" substrates of 3, 15 and 30 kPa for 1 d. Zoomed regions display details of F-actin organization in the apical region of the nucleus. Right: Corresponding quantification of the percentages of hMSCs having the actin cap (n = 3 experiments per group). **b** Left: Representative YAP images in control and cofilin-depleted hMSCs on "ON" substrates of 3, 15 and 30 kPa for 1 d. Right: Corresponding quantification of YAP nuc/cyto ratios (from left to right n = 64, 79, 68, 72, 85, 82 cells). Data are presented as mean ± s.e.m., and p values were obtained using one-way ANOVA followed by Tukey's post hoc test (**a**, **b**). Scale bars: 30 μm (**a**, **b**). Source data are provided as a Source Data file.

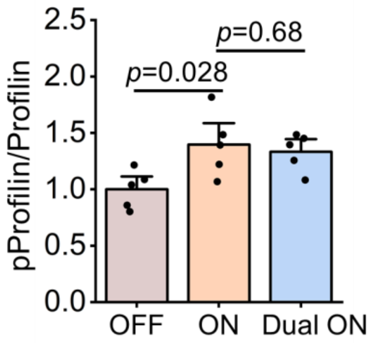

Supplementary Fig. 33 Effect of RGD/integrin and HAVDI/N-cadherin ligations on profilin phosphorylation. ELISA to quantify phosphorylation level of profilin in hMSCs on “OFF”, “ON”, “Dual ON” substrates for 1 d (n = 5 experiments per group). Data are presented as mean ± s.e.m., and p values were obtained using one-way ANOVA followed by Tukey’s post hoc test. Source data are provided as a Source Data file.

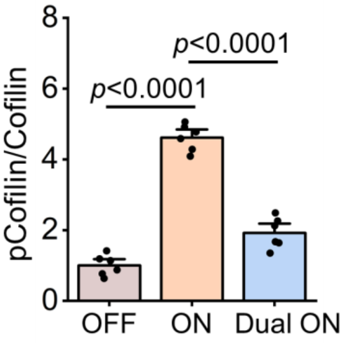

Supplementary Fig. 34 Effect of RGD/integrin and HAVDI/N-cadherin ligations on cofilin phosphorylation. ELISA to quantify phosphorylation level of cofilin in hMSCs on "OFF", "ON", "Dual ON" substrates for 1 d (n = 6 experiments per group). Data are presented as mean ± s.e.m., and p values were obtained using one-way ANOVA followed by Tukey's post hoc test. Source data are provided as a Source Data file.

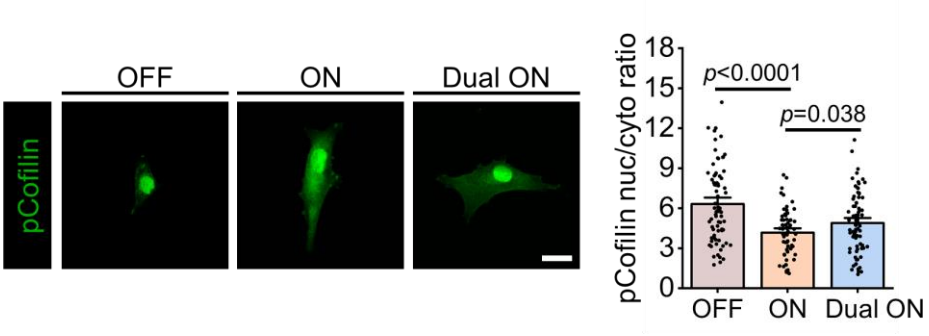

Supplementary Fig. 35 RGD/integrin and HAVDI/N-cadherin ligations regulate pCofilin nuclear localization. Left: Representative pCofilin images in hMSCs on "OFF", "ON" and "Dual ON" substrates for 1 d. Right: Corresponding quantification of pCofilin nuc/cyto ratios (from left to right n = 76, 63, 79 cells). Data are presented as mean ± s.e.m., and p values were obtained using one-way ANOVA followed by Tukey's post hoc test. Scale bars: 30 μm. Source data are provided as a Source Data file.

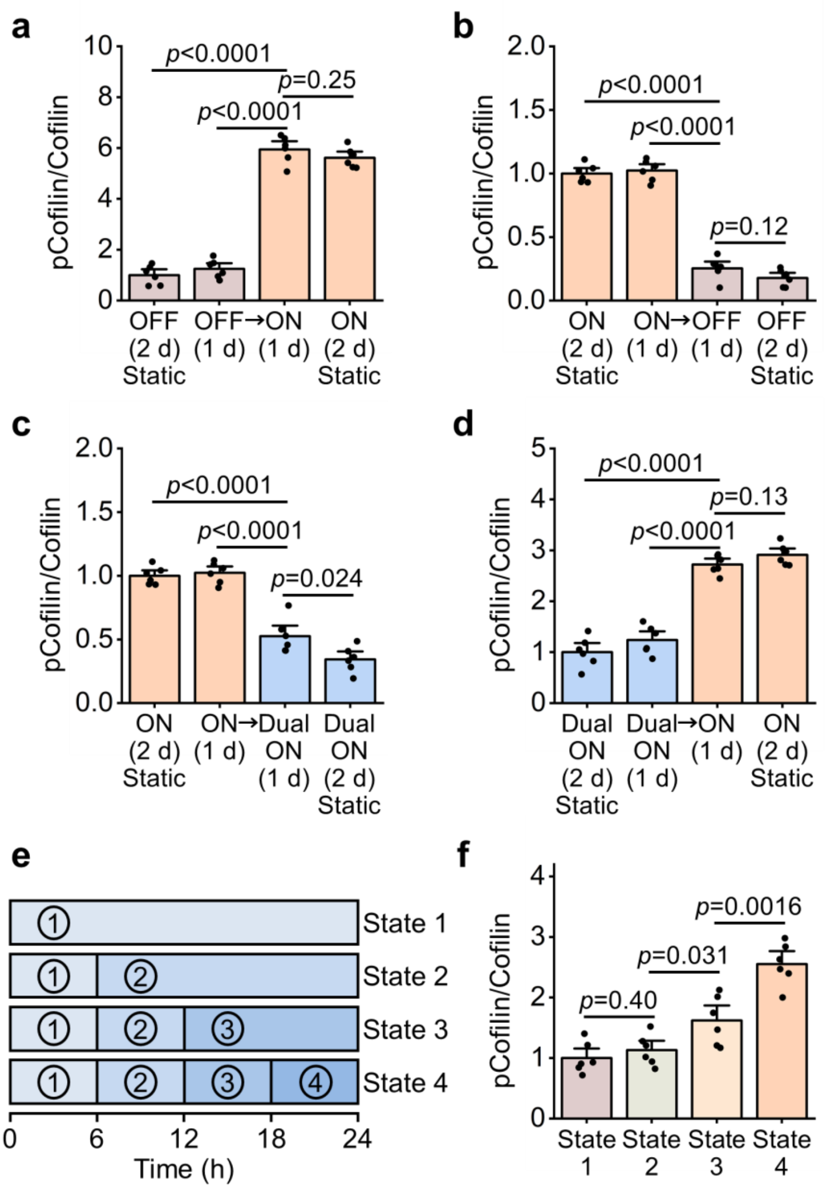

Supplementary Fig. 36 Effect of programmable RGD/integrin and HAVDI/N-cadherin ligations on cofilin phosphorylation. **a** ELISA to quantify phosphorylation level of cofilin in hMSCs for state switching from "OFF" for initial 1 d to "ON" for subsequent 1 d (n = 6 experiments per group). Cells were cultured on continuously "OFF" and "ON" substrates for 2 d as control groups. **b** ELISA to quantify phosphorylation level of cofilin in hMSCs for state switching from "ON" for initial 1 d to "OFF" for subsequent 1 d (n = 6 experiments per group). Cells were cultured on continuously "ON" and "OFF" substrates for 2 d as control groups. **c** ELISA to quantify phosphorylation level of cofilin in hMSCs for state switching from "ON" for initial 1 d to "Dual ON" for subsequent 1 d (n = 6 experiments per group). Cells were cultured on continuously "ON" and "Dual ON" substrates for 2 d as control groups. **d** ELISA to quantify phosphorylation level of

cofilin in hMSCs for state switching from "Dual ON" for initial 1 d to "ON" for subsequent 1 d (*n* = 6 experiments per group). Cells were cultured on continuously "Dual ON" and "ON" substrates for 2 d as control groups. **e** Concentrations of RGD and HAVDI were simultaneously varied every 6 h for the same conditions as **Fig. 3f**, until a total culture time of 24 h. **f** ELISA to quantify phosphorylation level of cofilin in hMSCs for the same conditions as **e** (*n* = 6 experiments per group). Data are presented as mean ± s.e.m., and *p* values were obtained using one-way ANOVA followed by Tukey's post hoc test (**a**-**d**, **f**). Source data are provided as a Source Data file.

Supplementary Table S1 Sequences of all DNA strands used. Name and corresponding sequences of all DNA strands used in this manuscript. Abbreviations: DS = displacement strand; SH = thiol; N_3 = azide; RF = rupture force. The subscript numbers after DS indicates the hybridizing length. The subscript S indicates that DNA_S is a scrambled sequence DNA.

Strand name	DNA sequence (5' to 3')
Primary strand 1	SH-ATATTGTTTGTTACACGGGATCCCGATTTT
DNA1 (RF: 56 pN)	N_3-ATTTGAAACGAAAATCGGGATCCCGTGTAA
DNA1 (RF: 12 pN)	AAAATCGGGATCCCGTGTAAATTTGAAACG-N_3
$DNA1_S$	N_3-TCAAGCTTAGATGTAACGAGACTAGGCATA
$DS1_{15}$	TTACACGGGATCCCG
$DS1_{20}$	TTACACGGGATCCCGATTTT
$DS1_{25}$ (*i.e.* DS1)	TTACACGGGATCCCGATTTTCGTTT
$DS1_{25S}$ (*i.e.* $DS1_S$)	ACACCTTGTCTTATTGTTCGCGGTA
$DS1_{30}$	TTACACGGGATCCCGATTTTCGTTTCAAAT
Primary strand 2	SH-GCGATACTCCTTTTCCGGAATTCCGCTTTT
DNA2 (RF: 56 pN)	N_3-TTAGATAGTAAAAAGCGGAATTCCGGAAAA
DNA2 (RF: 12 pN)	AAAAGCGGAATTCCGGAAAATTAGATAGTA-N_3
$DNA2_S$	N_3-ACTATGGTAGATCATAAAGAAACGAGTAAG
$DS2_{15}$	TTTTCCGGAATTCCG
$DS2_{20}$	TTTTCCGGAATTCCGCTTTT
$DS2_{25}$ (*i.e.* DS2)	TTTTCCGGAATTCCGCTTTTTACTA
$DS2_{25S}$ (*i.e.* $DS2_S$)	TTAATTTAGTCGTCTCCTCAGTCTT
$DS2_{30}$	TTTTCCGGAATTCCGCTTTTTACTATCTAA

Supplementary Table S2 Sequences of all peptides used. Name and corresponding sequences of all peptides used in this manuscript. Abbreviations: DBCO = dibenzocyclooctyne. The subscript S indicates that peptide$_S$ is a scrambled sequence peptide.

Peptide name	Peptide sequence
RGD	DBCO-GRGDSPK
FAM-RGD	DBCO-GRGDSPK-FAM
RGD$_S$	DBCO-GSDPRKG
HAVDI	HAVDIGGGK-DBCO
TAMRA-HAVDI	TAMRA-HAVDIGGGK-DBCO
HAVDI$_S$	AGVGDHIGK-DBCO
GFOGER	DBCO-GGYGGGP(GPP)$_5$GFOGER(GPP)$_5$GP
IKVAV	DBCO-GGASIKVAVS

Supplementary References

1. Freeman, R. *et al.* Instructing cells with programmable peptide DNA hybrids. *Nat. Commun.* **8**, 15982 (2017).
2. Arnold, M. *et al.* Activation of integrin function by nanopatterned adhesive interfaces. *ChemPhysChem* **5**, 383-388 (2004).
3. Huang, J. *et al.* Impact of order and disorder in RGD nanopatterns on cell adhesion. *Nano Lett.* **9**, 1111-1116 (2009).
4. Wang, X.F. & Ha, T. Defining single molecular forces required to activate integrin and notch signaling. *Science* **340**, 991-994 (2013).
5. Cocco, S., Monasson, R. & Marko, J.F. Force and kinetic barriers to unzipping of the DNA double helix. *Proc. Natl Acad. Sci. USA* **98**, 8608-8613 (2001).
6. De Gennes, P.G. Maximum pull out force on DNA hybrids. *C. R. Acad. Sci. Ser. IV Phys. Astrophys.* **2**, 1505-1508 (2001).
7. Hatch, K., Danilowicz, C., Coljee, V. & Prentiss, M. Demonstration that the shear force required to separate short double-stranded DNA does not increase significantly with sequence length for sequences longer than 25 base pairs. *Phys. Rev. E* **78**, 011920 (2008).
8. Krautbauer, R., Rief, M. & Gaub, H.E. Unzipping DNA oligomers. *Nano Lett.* **3**, 493-496 (2003).
