# Supplementary material for: Programmable integrin and N-cadherin adhesive interactions modulate mechanosensing of mesenchymal stem cells by cofilin phosphorylation
Source: Nat Commun. 2022 Nov 11;13:6854. doi: 10.1038/s41467-022-34424-0 (PMC9652405; doi:10.1038/s41467-022-34424-0)
Supplement: Supplementary file 2 — Description of Additional Supplementary Files [file 41467_2022_34424_MOESM2_ESM.pdf]

File name: Supplementary Movie 1

Description: Dynamic conjugation of TAMRA-labeled HAVDI. Live imaging of hMSCs on the same substrate for state switching from “ON” to “Dual ON”. Images were captured once every 2 minutes. Total time: 40 min. Scale bar: 100 μm.
