# Supplementary material for: Programmable integrin and N-cadherin adhesive interactions modulate mechanosensing of mesenchymal stem cells by cofilin phosphorylation
Source: Nat Commun. 2022 Nov 11;13:6854. doi: 10.1038/s41467-022-34424-0 (PMC9652405; doi:10.1038/s41467-022-34424-0)
Supplement: Supplementary file 4 — Reporting Summary [file 41467_2022_34424_MOESM4_ESM.pdf]

nature portfolio

Corresponding author(s): Min Lin

Last updated by author(s): Oct 22, 2022

Reporting Summary

Nature Portfolio wishes to improve the reproducibility of the work that we publish. This form provides structure for consistency and transparency in reporting. For further information on Nature Portfolio policies, see our Editorial Policies and the Editorial Policy Checklist.

Statistics

For all statistical analyses, confirm that the following items are present in the figure legend, table legend, main text, or Methods section.

n/a	Confirmed	
☐	☒	The exact sample size (*n*) for each experimental group/condition, given as a discrete number and unit of measurement
☐	☒	A statement on whether measurements were taken from distinct samples or whether the same sample was measured repeatedly
☐	☒	The statistical test(s) used AND whether they are one- or two-sided<br>*Only common tests should be described solely by name; describe more complex techniques in the Methods section.*
☒	☐	A description of all covariates tested
☐	☒	A description of any assumptions or corrections, such as tests of normality and adjustment for multiple comparisons
☐	☒	A full description of the statistical parameters including central tendency (e.g. means) or other basic estimates (e.g. regression coefficient) AND variation (e.g. standard deviation) or associated estimates of uncertainty (e.g. confidence intervals)
☐	☒	For null hypothesis testing, the test statistic (e.g. *F*, *t*, *r*) with confidence intervals, effect sizes, degrees of freedom and *P* value noted<br>*Give P values as exact values whenever suitable.*
☒	☐	For Bayesian analysis, information on the choice of priors and Markov chain Monte Carlo settings
☒	☐	For hierarchical and complex designs, identification of the appropriate level for tests and full reporting of outcomes
☒	☐	Estimates of effect sizes (e.g. Cohen's *d*, Pearson's *r*), indicating how they were calculated

Our web collection on statistics for biologists contains articles on many of the points above.

Software and code

Policy information about availability of computer code

Data collection	Confocal images were acquired with the Olympus FV31S-SW (2.3.1.163) software on Olympus FV3000 microscope.
Data analysis	Data analysis was performed with the assistance of Origin (2020), Matlab (R2014a), Bitplane Imaris (7.2.3), and Image J (1.52p).

For manuscripts utilizing custom algorithms or software that are central to the research but not yet described in published literature, software must be made available to editors and reviewers. We strongly encourage code deposition in a community repository (e.g. GitHub). See the Nature Portfolio guidelines for submitting code & software for further information.

Data

Policy information about availability of data

All manuscripts must include a data availability statement. This statement should provide the following information, where applicable:

- Accession codes, unique identifiers, or web links for publicly available datasets
- A description of any restrictions on data availability
- For clinical datasets or third party data, please ensure that the statement adheres to our policy

All data in this study are available in the manuscript and the Supplementary materials or from the corresponding author upon reasonable request. Source data are provided with this paper.

Field-specific reporting

Please select the one below that is the best fit for your research. If you are not sure, read the appropriate sections before making your selection.

☒ Life sciences ☐ Behavioural & social sciences ☐ Ecological, evolutionary & environmental sciences

For a reference copy of the document with all sections, see nature.com/documents/nr-reporting-summary-flat.pdf

Life sciences study design

All studies must disclose on these points even when the disclosure is negative.

Sample size	No statistical methods were used to pre-determine sample sizes, which were chosen based on previous experience with these metrics. For all statistical analyses, three biological replicates was chosen as a self-imposed minimum. The exact replication numbers, sample sizes and statistical methods are described in detail in the text.
Data exclusions	The data of cells having contact with neighbors were excluded for analysis in our study.
Replication	All experiments were repeated successfully 3+ times to ensure reproducibility.
Randomization	All samples were randomly allocated into experimental groups.
Blinding	Investigators were blinded to group allocation during data collection and analysis.

Reporting for specific materials, systems and methods

We require information from authors about some types of materials, experimental systems and methods used in many studies. Here, indicate whether each material, system or method listed is relevant to your study. If you are not sure if a list item applies to your research, read the appropriate section before selecting a response.

Materials & experimental systems

n/a	Involved in the study	
☐	☒	Antibodies
☐	☒	Eukaryotic cell lines
☒	☐	Palaeontology and archaeology
☒	☐	Animals and other organisms
☒	☐	Human research participants
☒	☐	Clinical data
☒	☐	Dual use research of concern

Methods

n/a	Involved in the study	
☒	☐	ChIP-seq
☒	☐	Flow cytometry
☒	☐	MRI-based neuroimaging

Antibodies

Antibodies used	All antibodies used in this study are listed as follows:<br>1. anti-Integrin β1 (1:500, mouse, Abcam, ab30394)<br>2. anti-N-Cadherin (1:200, rabbit, Cell Signaling, 13116)<br>3. anti-YAP (1:100, rabbit, Cell Signaling, 14074)<br>4. anti-RUNX2 (1:1000, rabbit, Cell Signaling, 12556)<br>5. anti-Paxillin (1:200, rabbit, Abcam, ab32084)<br>6. anti-Lamin A/C (1:100, mouse, Cell Signaling, 4777)<br>7. anti-Phospho-Cofilin (1:100, rabbit, Cell Signaling, 3313)<br>8. anti-Lamin A (1:100, mouse, Cell Signaling, 86846)<br>9. AlexaFluor-488[H+L] secondary antibodies (1:500, goat anti-rabbit, Cell Signaling, 4412)<br>10. AlexaFluor-647[H+L] secondary antibodies (1:500, goat anti-mouse, Cell Signaling, 4410)
Validation	All the antibodies were used according to the manufacturer's protocol.<br>1. anti-Integrin β1 (1:500, mouse, Abcam, ab30394)<br>https://www.abcam.cn/integrin-beta-1-antibody-12g10-ab30394.html<br>2. anti-N-Cadherin (1:200, rabbit, Cell Signaling, 13116)<br>https://www.cellsignal.cn/products/primary-antibodies/n-cadherin-d4r1h-xp-rabbit-mab/13116?site-search-type=Products&N=4294956287&Ntt=13116&fromPage=plp&_requestid=1174379<br>3. anti-YAP (1:100, rabbit, Cell Signaling, 14074)<br>https://www.cellsignal.cn/products/primary-antibodies/yap-d8h1x-xp-rabbit-mab/14074?site-search-type=Products&N=4294956287&Ntt=14074&fromPage=plp&_requestid=1174676<br>4. anti-RUNX2 (1:1000, rabbit, Cell Signaling, 12556)<br>https://www.cellsignal.cn/products/primary-antibodies/runx2-d1l7f-rabbit-mab/12556?site-search-

type=Products&N=4294956287&Ntt=12556&fromPage=plp&_requestid=1175556
5. anti-Paxillin (1:200, rabbit, Abcam, ab32084)
https://www.abcam.cn/paxillin-antibody-y113-ab32084.html
6. anti-Lamin A/C (1:100, mouse, Cell Signaling, 4777)
https://www.cellsignal.cn/products/primary-antibodies/lamin-a-c-4c11-mouse-mab/4777?site-search-type=Products&N=4294956287&Ntt=4777&fromPage=plp&_requestid=1176209
7. anti-Phospho-Cofilin (1:100, rabbit, Cell Signaling, 3313)
https://www.cellsignal.cn/products/primary-antibodies/phospho-cofilin-ser3-77g2-rabbit-mab/3313?site-search-type=Products&N=4294956287&Ntt=3313&fromPage=plp&_requestid=1176411
8. anti-Lamin A (1:100, mouse, Cell Signaling, 86846)
https://www.cellsignal.cn/products/primary-antibodies/lamin-a-133a2-mouse-mab/86846?site-search-type=Products&N=4294956287&Ntt=86846&fromPage=plp&_requestid=1176560
9. AlexaFluor-488[H+L] secondary antibodies (1:500, goat anti-rabbit, Cell Signaling, 4412)
https://www.cellsignal.cn/products/secondary-antibodies/anti-rabbit-igg-h-l-f-ab-2-fragment-alexa-fluor-488-conjugate/4412?site-search-type=Products&N=4294956287&Ntt=4412&fromPage=plp&_requestid=1172382
10. AlexaFluor-647[H+L] secondary antibodies (1:500, goat anti-mouse, Cell Signaling, 4410)
https://www.cellsignal.cn/products/secondary-antibodies/anti-mouse-igg-h-l-f-ab-2-fragment-alexa-fluor-647-conjugate/4410?site-search-type=Products&N=4294956287&Ntt=4410&fromPage=plp&_requestid=1172519

Eukaryotic cell lines

Policy information about cell lines

Cell line source(s)	Human mesenchymal stem cells were isolated from human bone marrow provided by a commercial source (Cyagen Biosciences).
Authentication	Cell lines were authenticated by Cyagen Biosciences.
Mycoplasma contamination	Cell lines used in these studies tested negative for mycoplasma contamination.
Commonly misidentified lines (See ICLAC register)	No commonly misidentified cell lines were used.
